# Supplementary material for: The molecular landscape of Asian breast cancers reveals clinically relevant population-specific differences
Source: Nat Commun. 2020 Dec 22;11:6433. doi: 10.1038/s41467-020-20173-5 (PMC7755902; doi:10.1038/s41467-020-20173-5)
Supplement: Supplementary file 1 — Supplementary Information [file 41467_2020_20173_MOESM1_ESM.pdf]

The Molecular Landscape of Asian Breast Cancers Reveals Clinically Relevant Population-Specific Differences

Pan, JW et al.

Supplementary Information

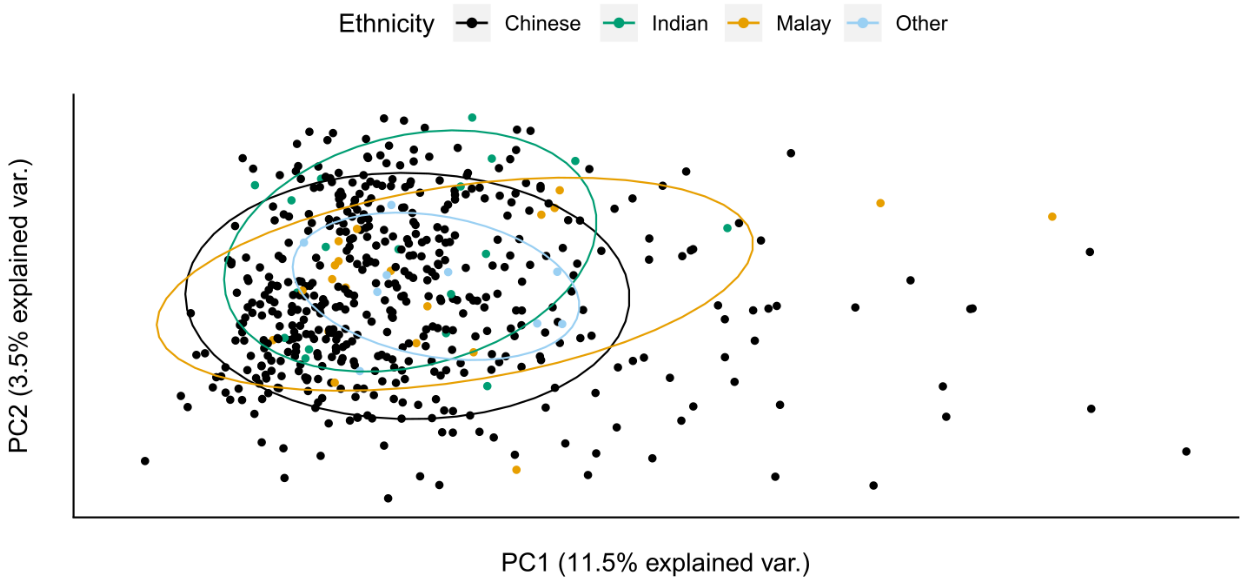

Supplementary Figure 1. Ethnicity and gene expression in MyBrCa breast tumours. Principal component analysis of gene expression in MyBrCa tumours showing the overlap between different ethnicities (n: Chinese=498, Indian=19, Malay=26, Other=9).

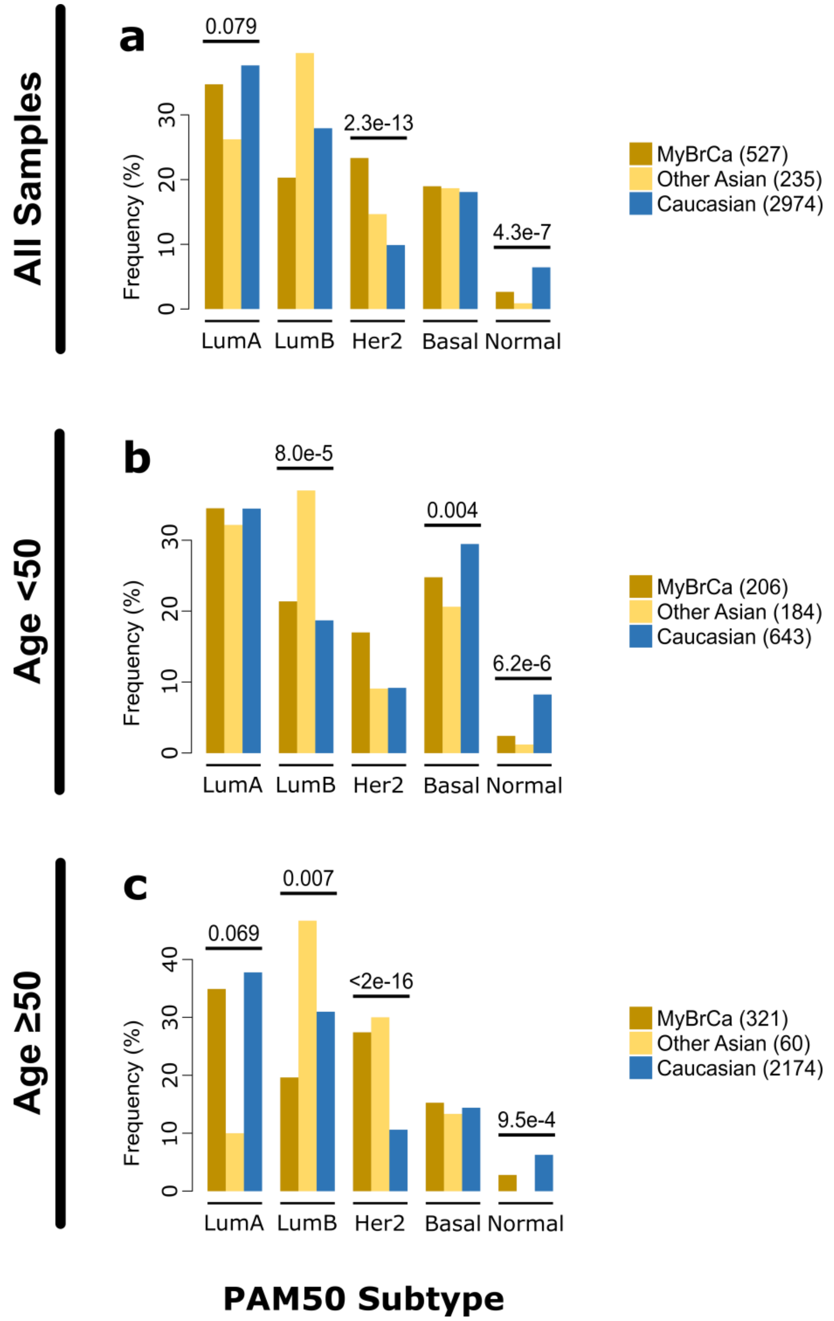

Supplementary Figure 2. Molecular subtypes of Malaysian breast cancer. Comparison of PAM50 molecular subtype distribution across Malaysian (MyBrCa), other Asian (Korean[1], TCGA[2] Asian), and Caucasian (TCGA[2] Caucasian, METABRIC[3], Nik-Zainal 2016[4]) cohorts. Comparisons were done using the full cohorts **(a)** as well as with only patients below **(b)** or above **(c)** the age of 50 as a rough proxy for menopausal status. Numbers above the bars are p-values denoting significant differences between Asians and Caucasians for that subtype, as determined by Pearson's chi-square test. Numbers in the figure legend indicate sample size.

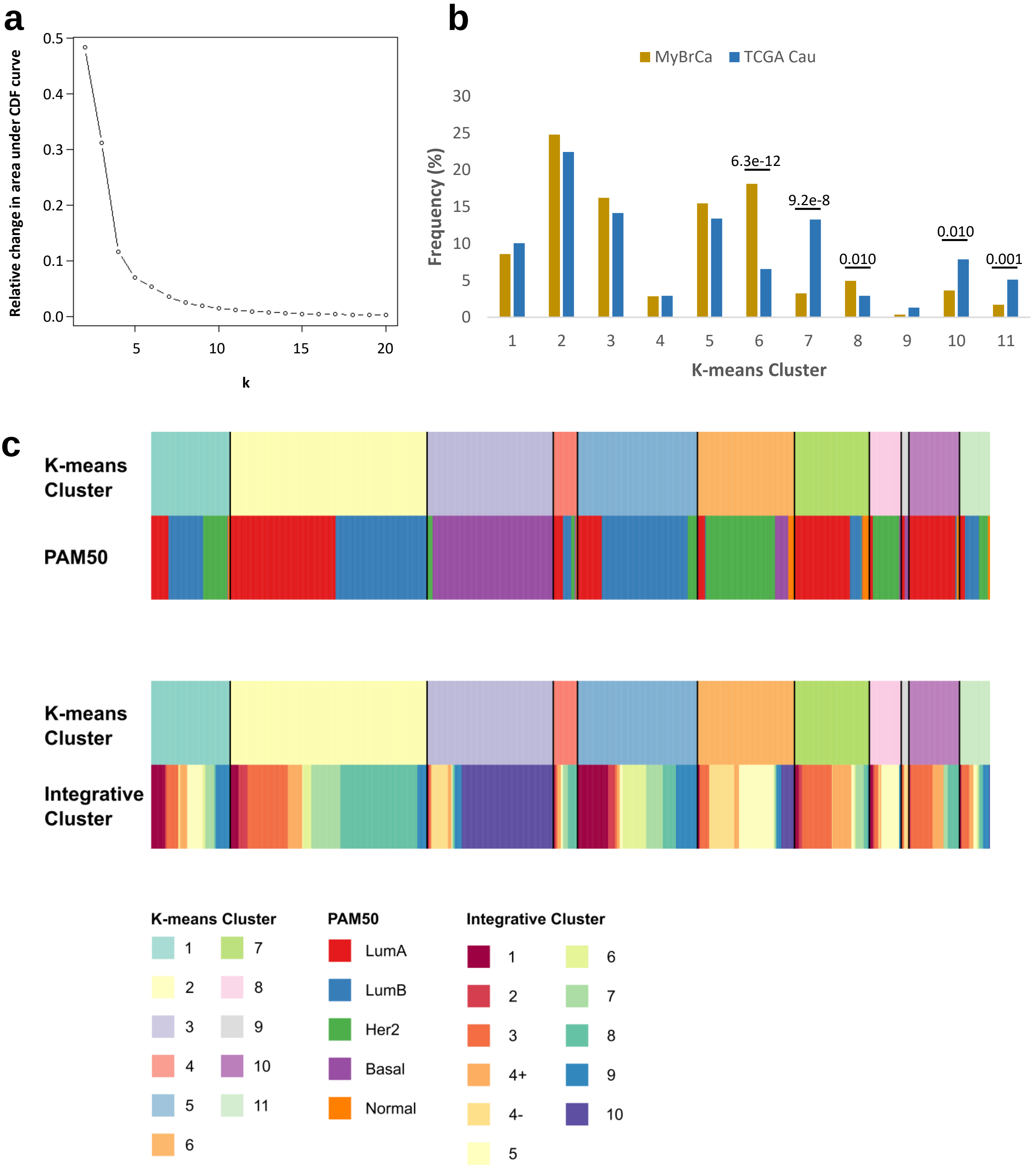

Supplementary Figure 3. Unsupervised clustering of MyBrCa and TCGA Caucasian samples. K-means consensus clustering of MyBrCa and TCGA Caucasian gene expression data in order to determine the existence of exclusive Asian or Caucasian subtypes. **(a)** Plot of the relative change in area under the empirical cumulative distribution (CDF) of a consensus matrix, used to determine that the maximum number of meaningful clusters in our data is k=11. **(b)** Frequency of samples in each k-means cluster by cohort when k=11. P-values are for Pearson's chi-square test. P-values above 0.1 are not shown. **(c)** Comparison of the 11 k-means clusters to PAM50 and Integrative Clusters.

a

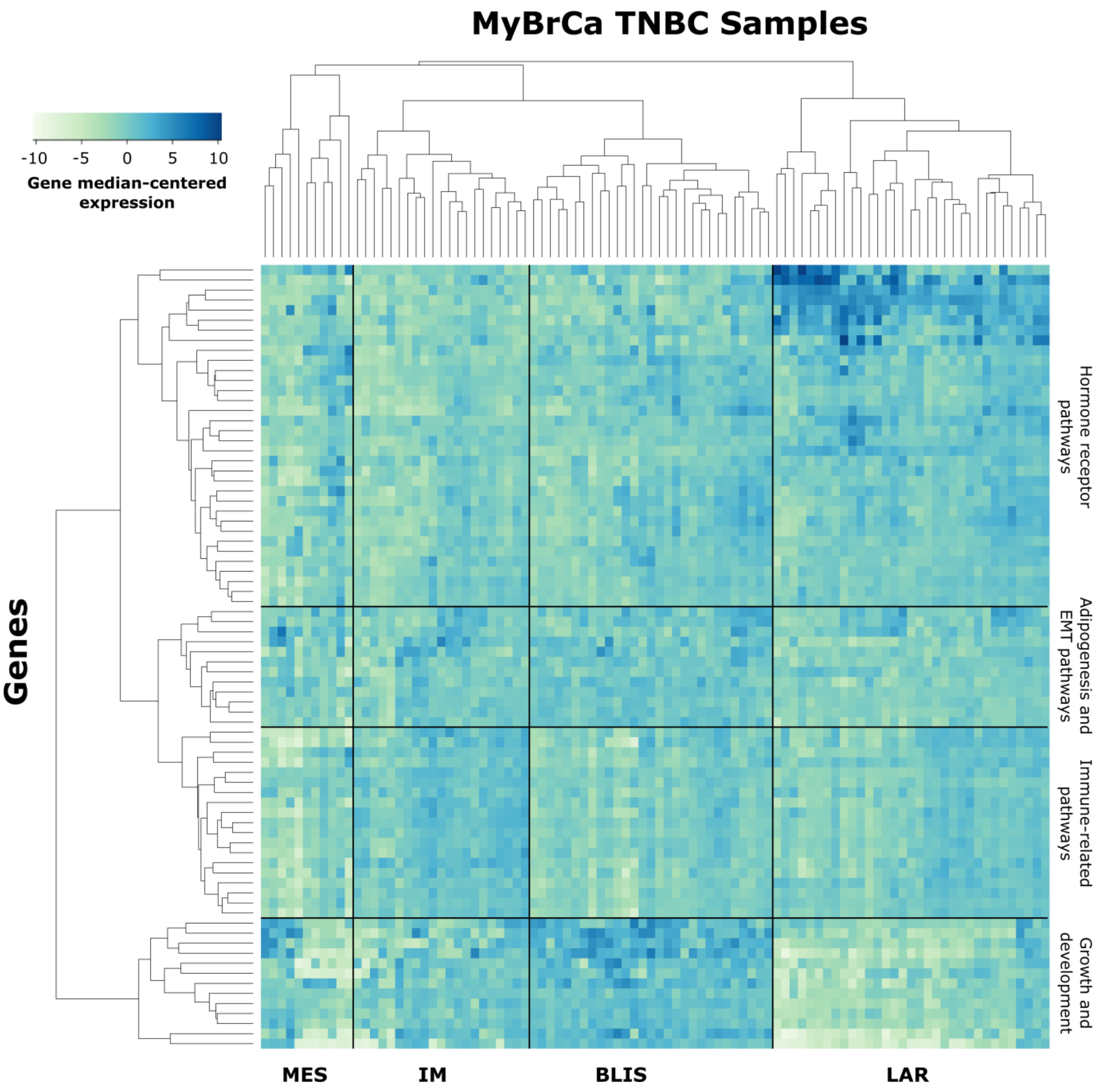

b

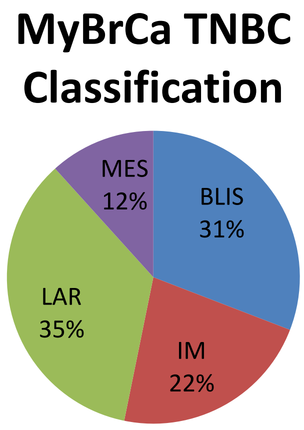

Supplementary Figure 4. Classification of MyBrCa TNBC samples. (a) Heatmap of MyBrCa TNBC samples (n=94) showing the classification of samples into mesenchymal (MES), immuno-modulatory (IM), basal-like immuno-suppressed (BLIS) and luminal androgen receptor (LAR) subtypes, using the 80-gene signature from Burstein et al. (2015)[5] on gene median-centered transcript per million (TPM) expression scores. **(b)** Pie chart showing the proportion of MyBrCa TNBC samples that were classified into each subtype.

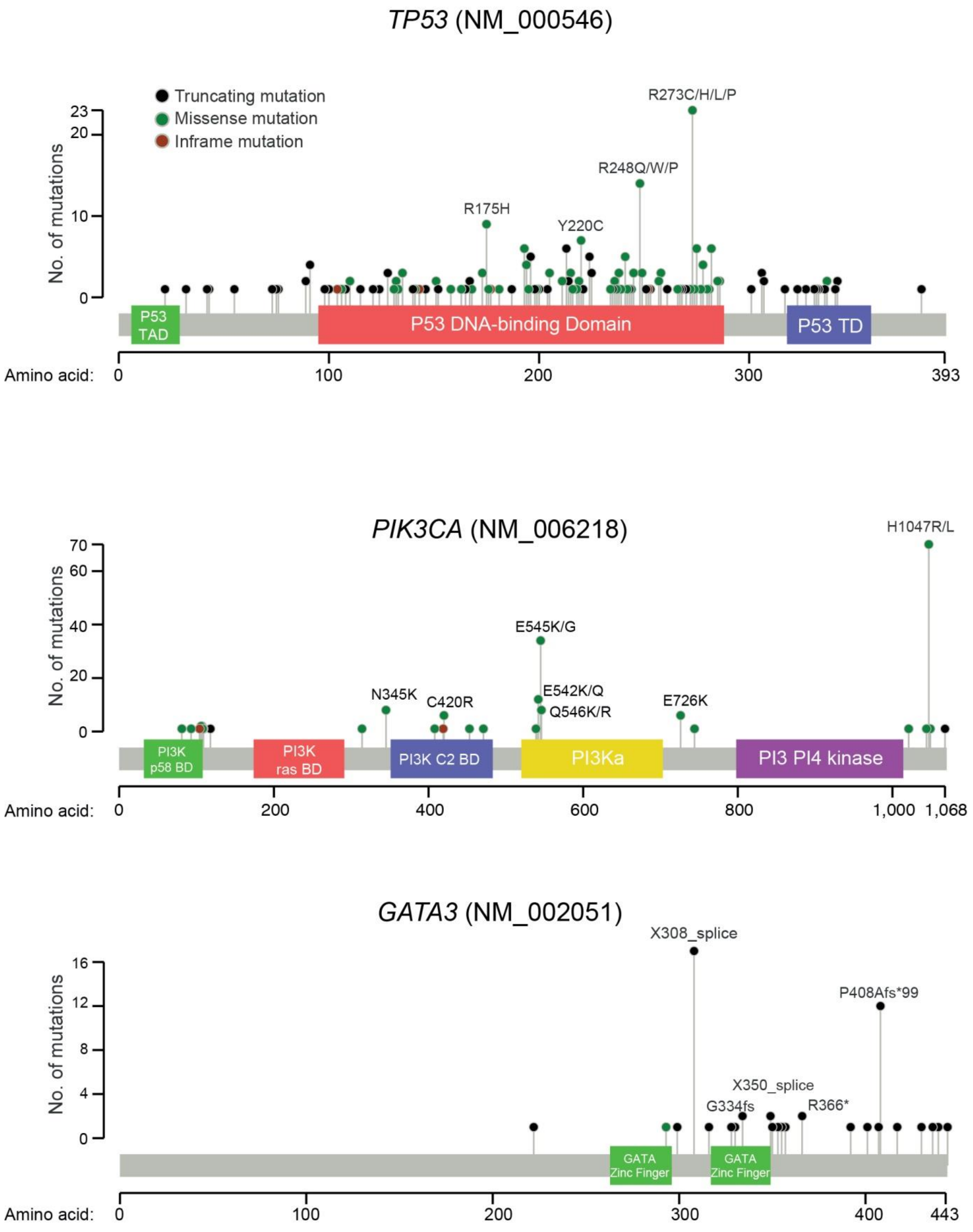

Supplementary Figure 5. Somatic mutation positions of *TP53*, *PIK3CA* and *GATA3* in MyBrCa. Lollipop plots depicting positions of truncating, missense and inframe somatic mutations, as well as the most prominent positions in each gene.

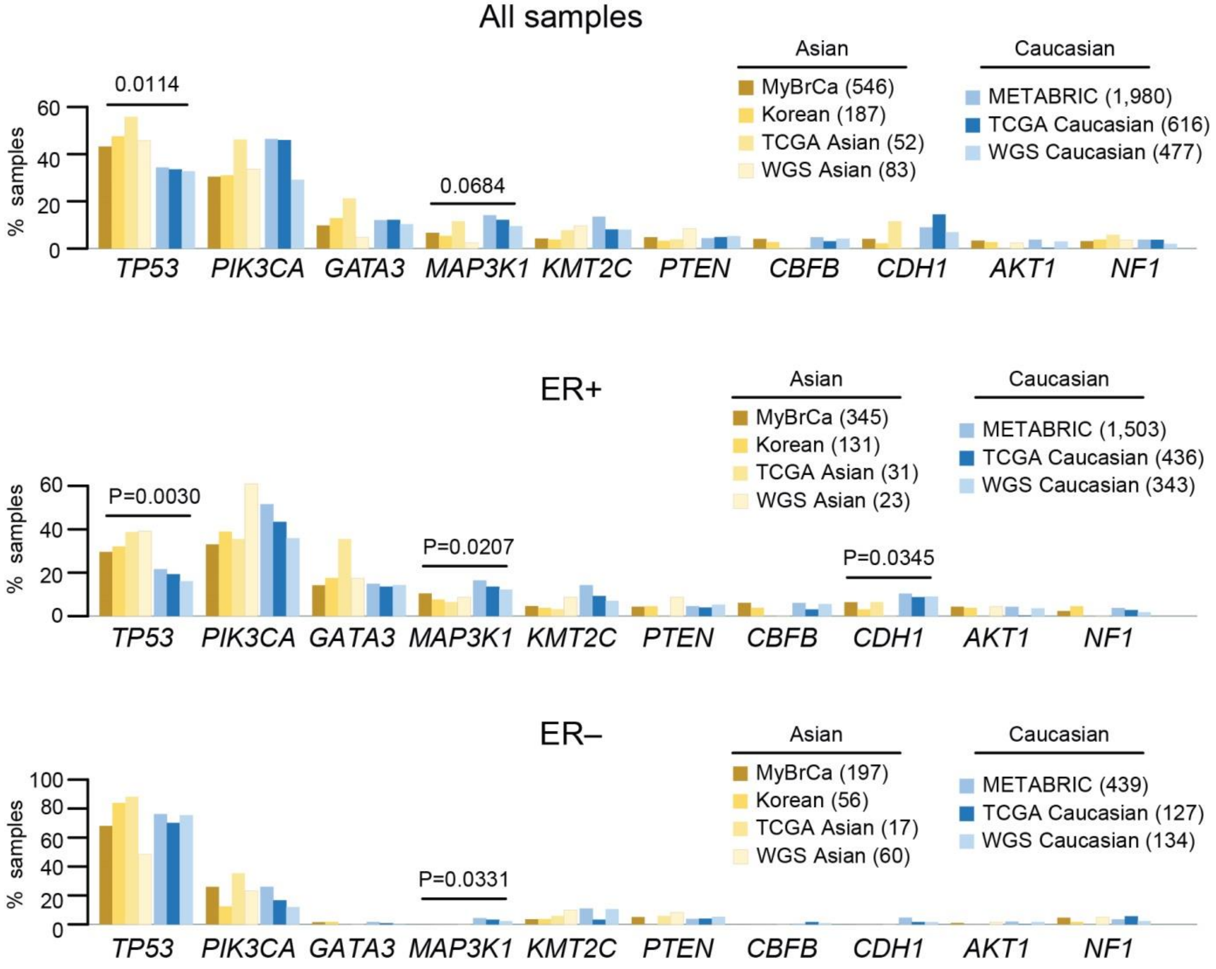

Supplementary Figure 6. Mutational prevalence of main breast cancer genes in Asian and Caucasian breast tumours, separated by ER status. Comparison of mutational prevalence of main breast cancer genes in Asian (MyBrCa, Korean, TCGA Asian, WGS Asian) and Caucasian (METABRIC, TCGA Caucasian, WGS Caucasian) breast tumours, in all samples (top), or separated according to their ER status (middle and bottom). P-values from 2-sided Student's *t*-test comparing Asian versus Caucasian samples.

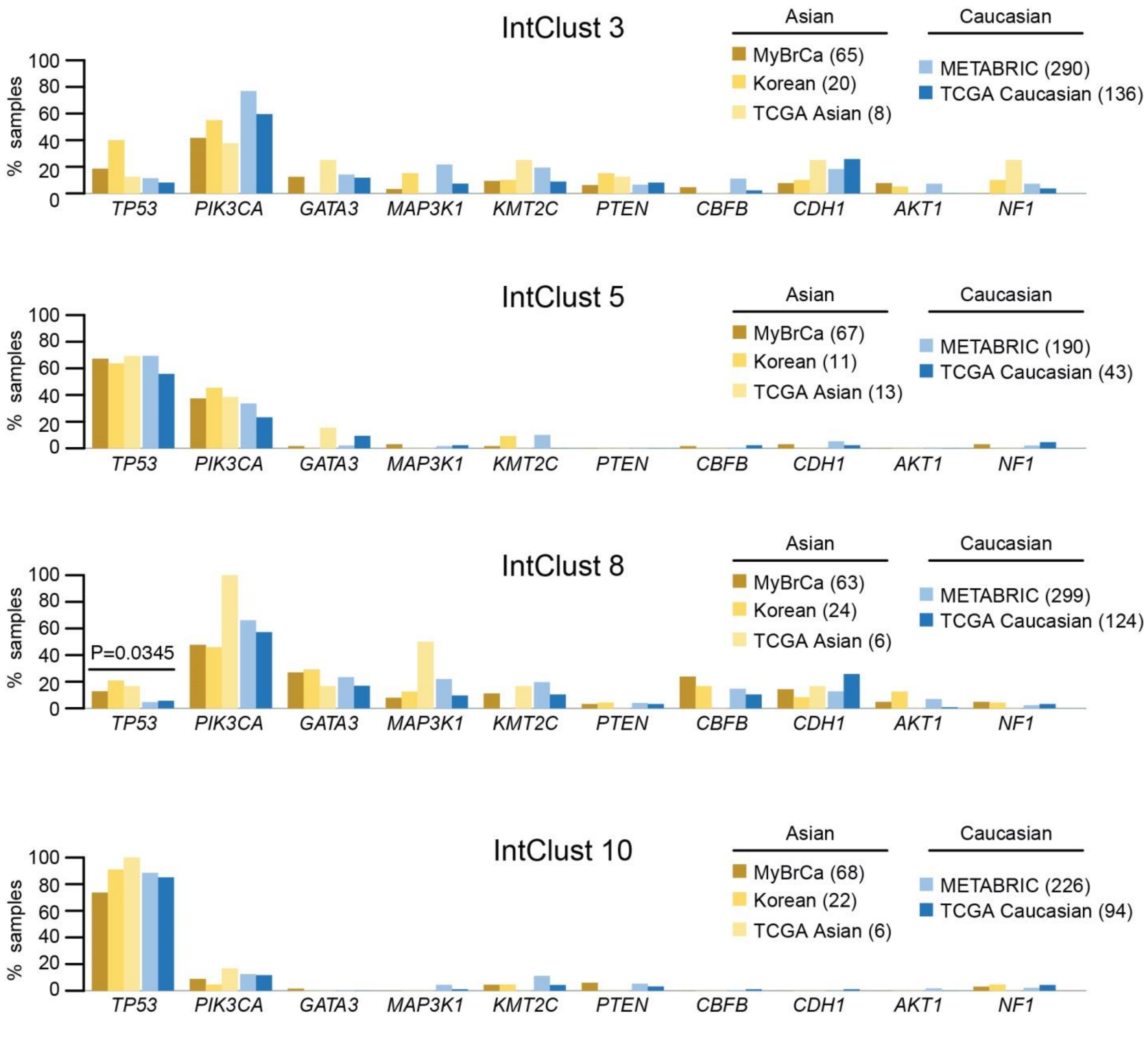

Supplementary Figure 7. Mutational prevalence of main breast cancer genes in Asian and Caucasian breast tumours, separated by IntClust. Comparison of mutational prevalence of main breast cancer genes in Asian and Caucasian, limited to all samples from Integrative Clusters 3,5, 8 and 10 – these IntClusts are with the most samples overall to permit meaningful cross comparison. P-value from 2-sided Student's *t*-test comparing Asian versus Caucasian samples.

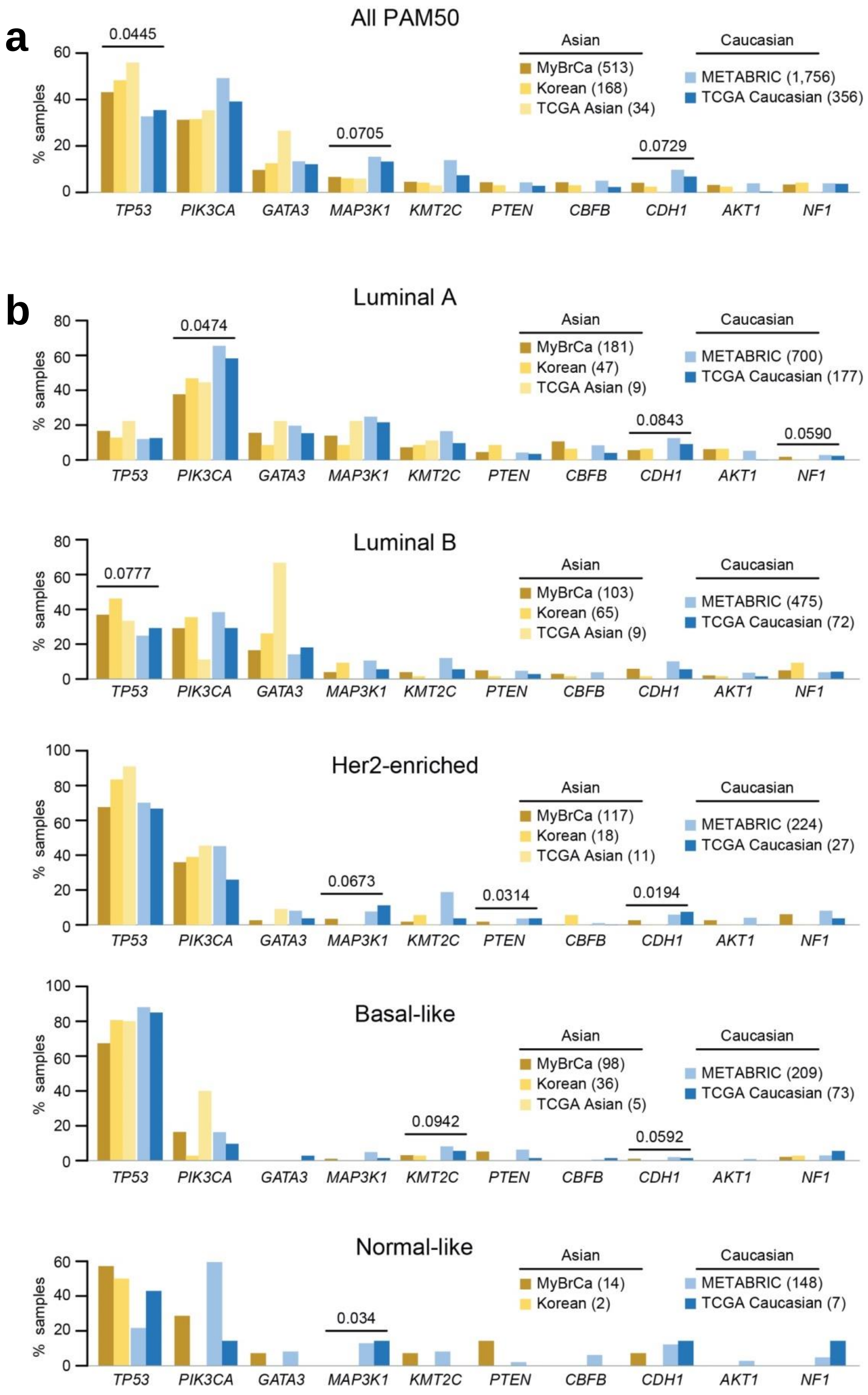

Supplementary Figure 8. Mutational prevalence of main breast cancer genes in Asian and Caucasian breast tumours, separated by PAM50. Comparison of mutational prevalence of main breast cancer genes in Asian and Caucasian, limited to all samples with available PAM50 classification **(a)** and to PAM50 subtypes **(b)**. P-values from 2-sided Student's *t*-test comparing Asian versus Caucasian samples.

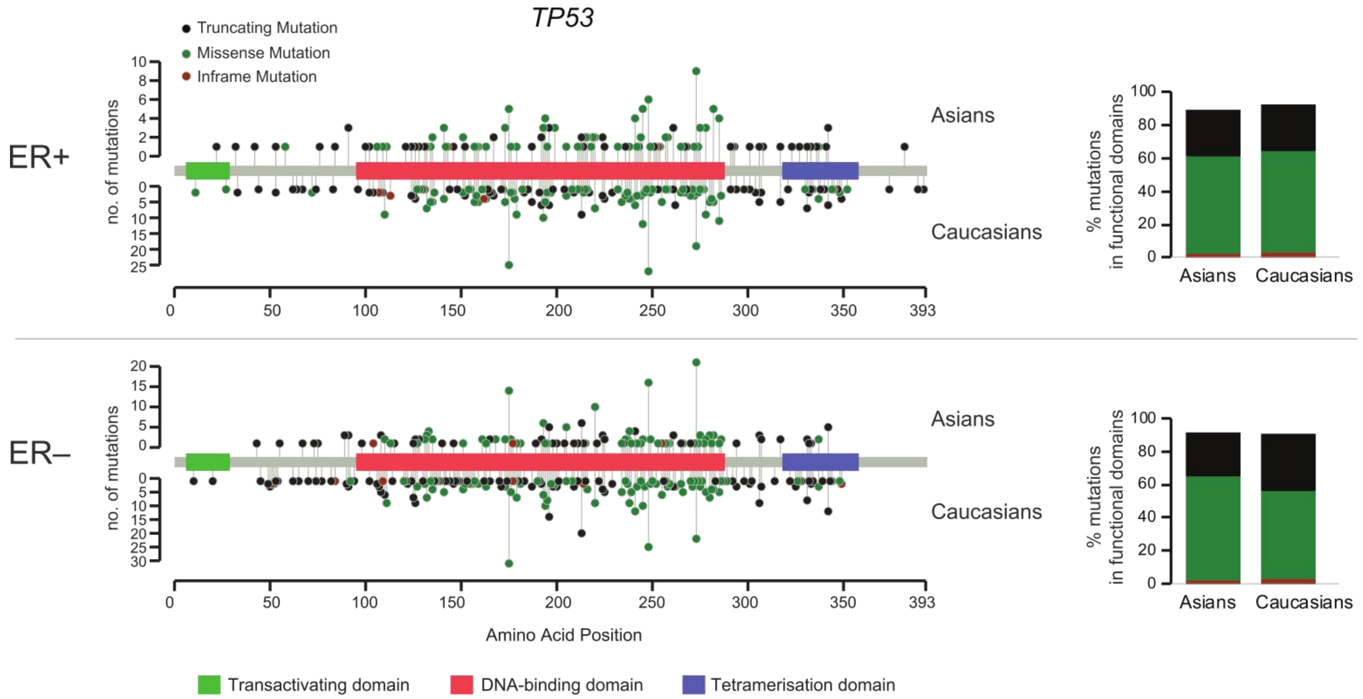

Supplementary Figure 9. Comparison of *TP53* somatic mutations in Asians and Caucasians. Positions of *TP53* mutations in ER+ and ER– samples, separated by ethnicity. Right: Percent of mutations in each category that are located in transactivating, DNA-binding, or tetramisation domains of *TP53*.

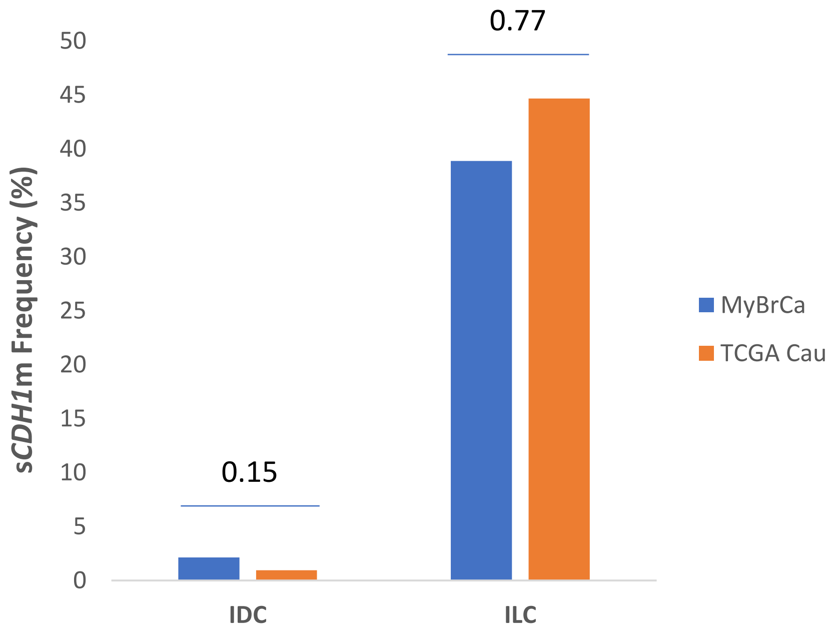

Supplementary Figure 10. Prevalence of *CDH1* somatic mutations in different histological subtypes. Comparison of prevalence of somatic mutations in the *CDH1* gene in the MyBrCa and TCGA Caucasian cohorts, stratified by histological subtypes (IDC: Invasive ductal carcinoma; ILC: Invasive lobular carcinoma). The absolute values for IDC are MyBrCa: 11/515 (2.1%) versus TCGA Cau.: 4/424 (0.9%), while the absolute values for ILC are MyBrCa: 7/18 (39%) vs TCGA Cau.: 67/150 (44.7%). P-values shown are determined by chi-square test.

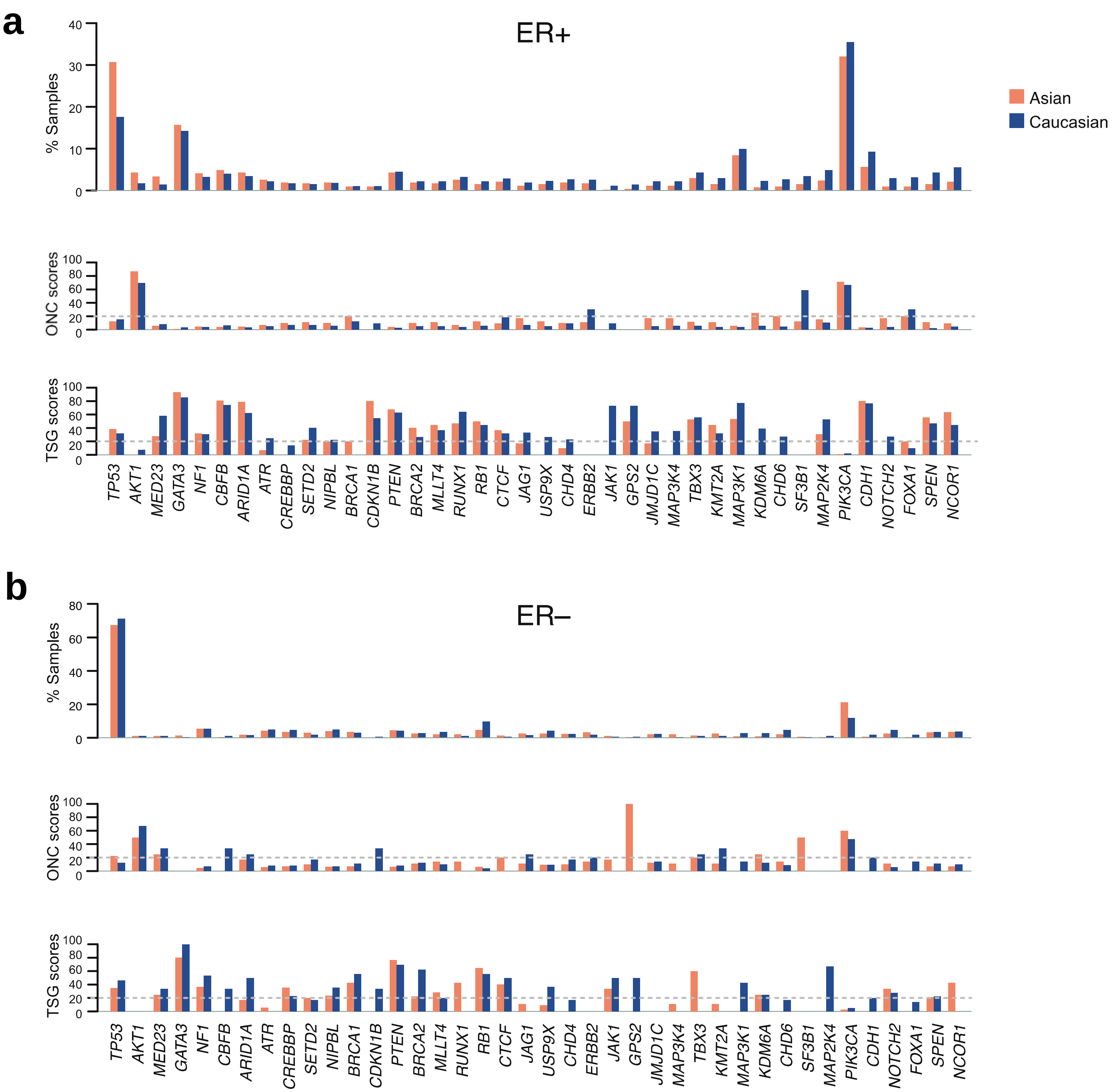

Supplementary Figure 11. Driver genes in Asian and Caucasian breast tumours. Bar plots depict mutation rates of driver genes (top panels), as well as ONC (middle) and TSG (bottom) scores in Asian or Caucasian samples, separated into ER+ **(a)** or ER– **(b)**. Dotted grey lines are at 20 ONC/TSG scores.

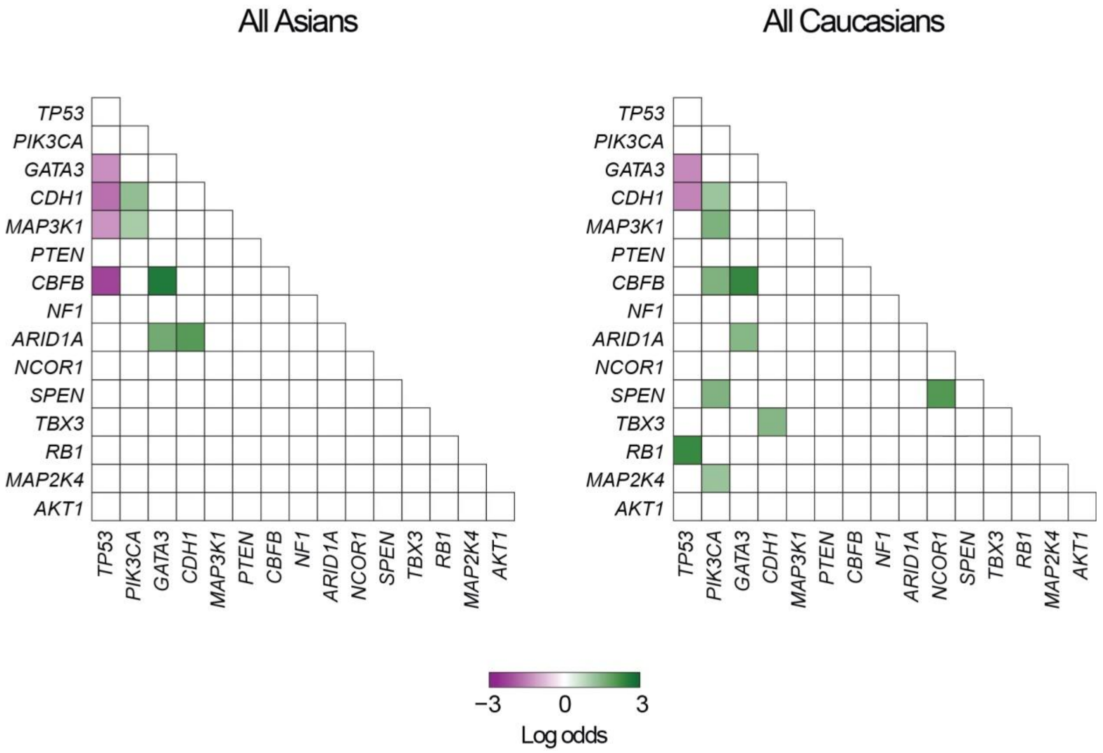

Supplementary Figure 12. Association patterns of driver genes in Asian and Caucasian breast tumours. Heat maps depicting co-occurrence (green) or mutual exclusivity (purple) of somatic SNV or indel mutations in all Asian (left) and Caucasian (right) breast tumours.

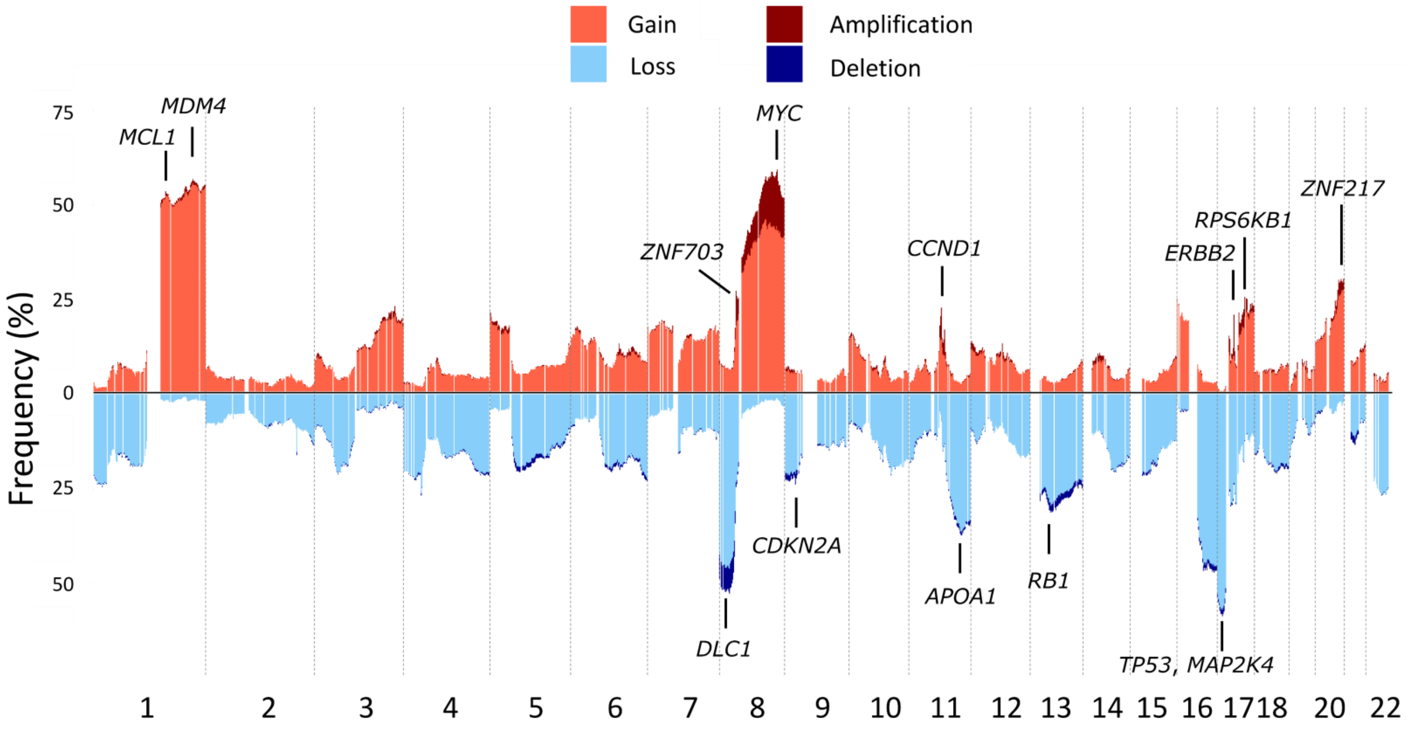

Supplementary Figure 13. Copy number profile of MyBrCa tumours from shallow WGS. Frequency of copy-number amplifications, gain, loss, and deletions are based on 100kb-window segmented calls, as called by QDNASeq.

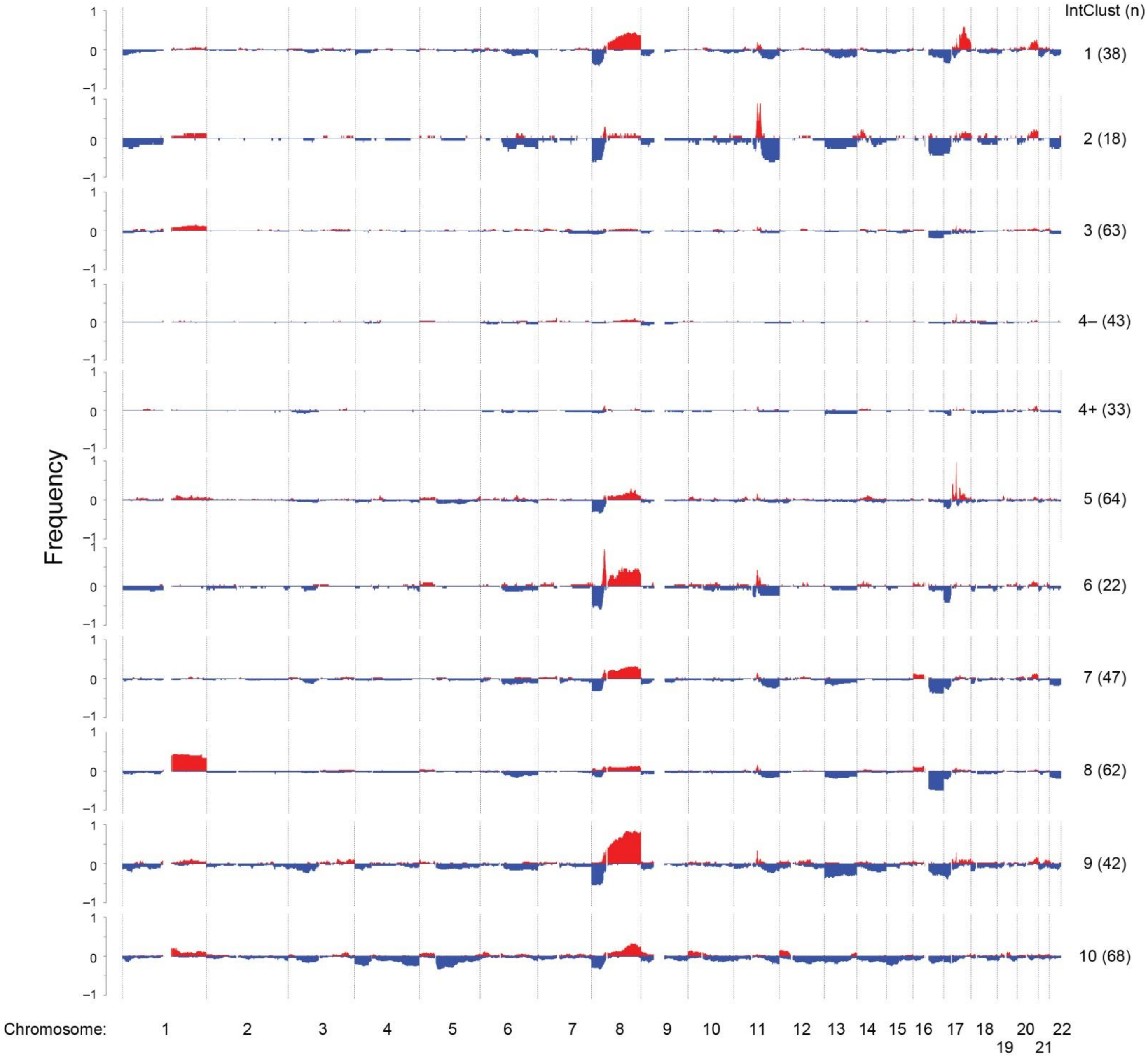

Supplementary Figure 14. Copy number aberration (CNA) of MyBrCa samples. Copy number aberration plots depicting the frequencies of CNAs in each IntClust.

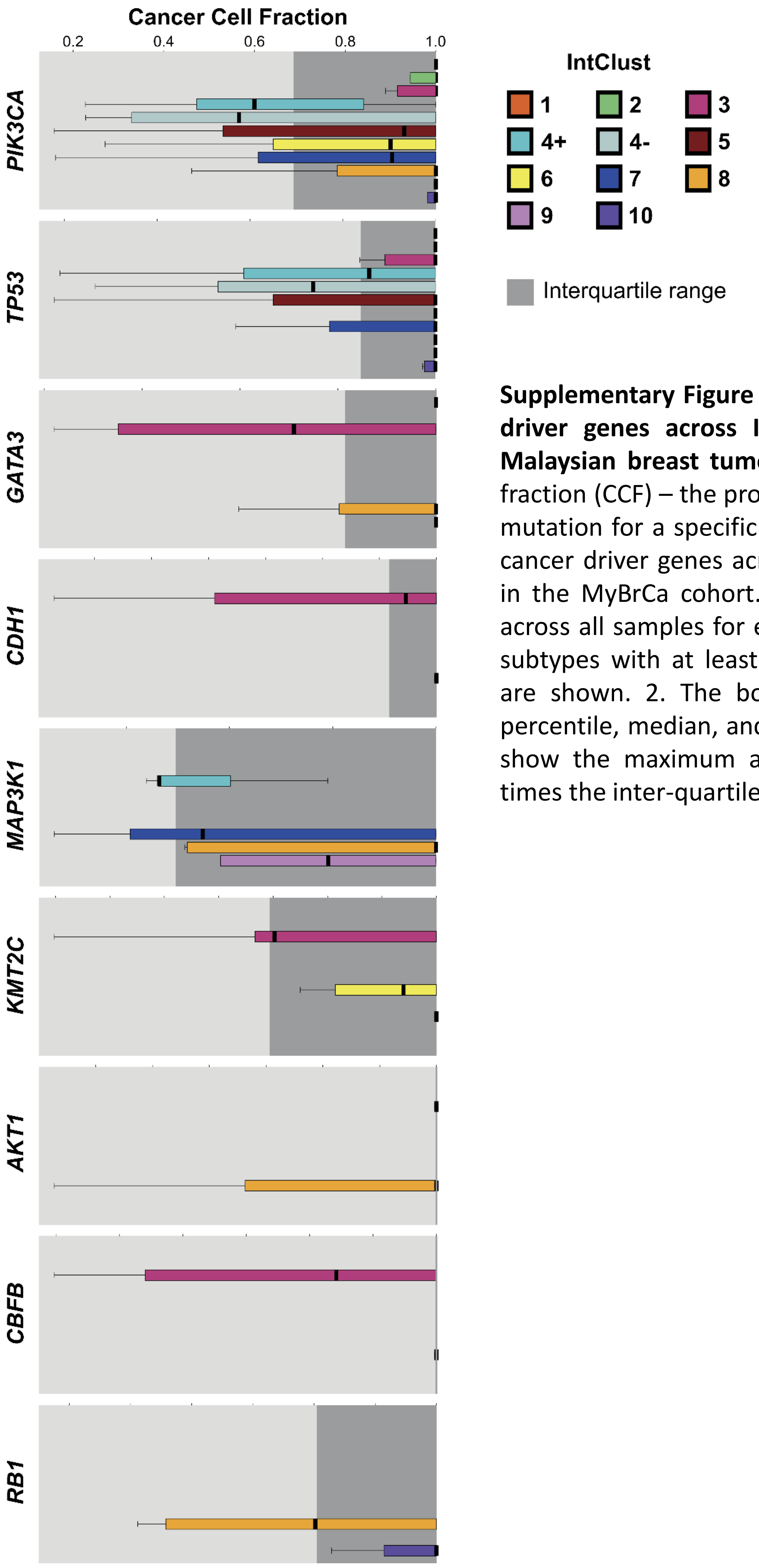

Supplementary Figure 15. Cancer cell fraction of nine driver genes across Integrative Cluster subtypes in Malaysian breast tumours. Comparison of cancer cell fraction (CCF) – the proportion of cancer cells carrying a mutation for a specific gene – for nine common breast cancer driver genes across Integrative Cluster subtypes in the MyBrCa cohort. The interquartile range of CCF across all samples for each gene is shown in grey. Only subtypes with at least three samples in that category are shown. 2. The boxes in box plots indicate 25th percentile, median, and 75th percentile, while whiskers show the maximum and minimum values within 1.5 times the inter-quartile range from the edge of the box.

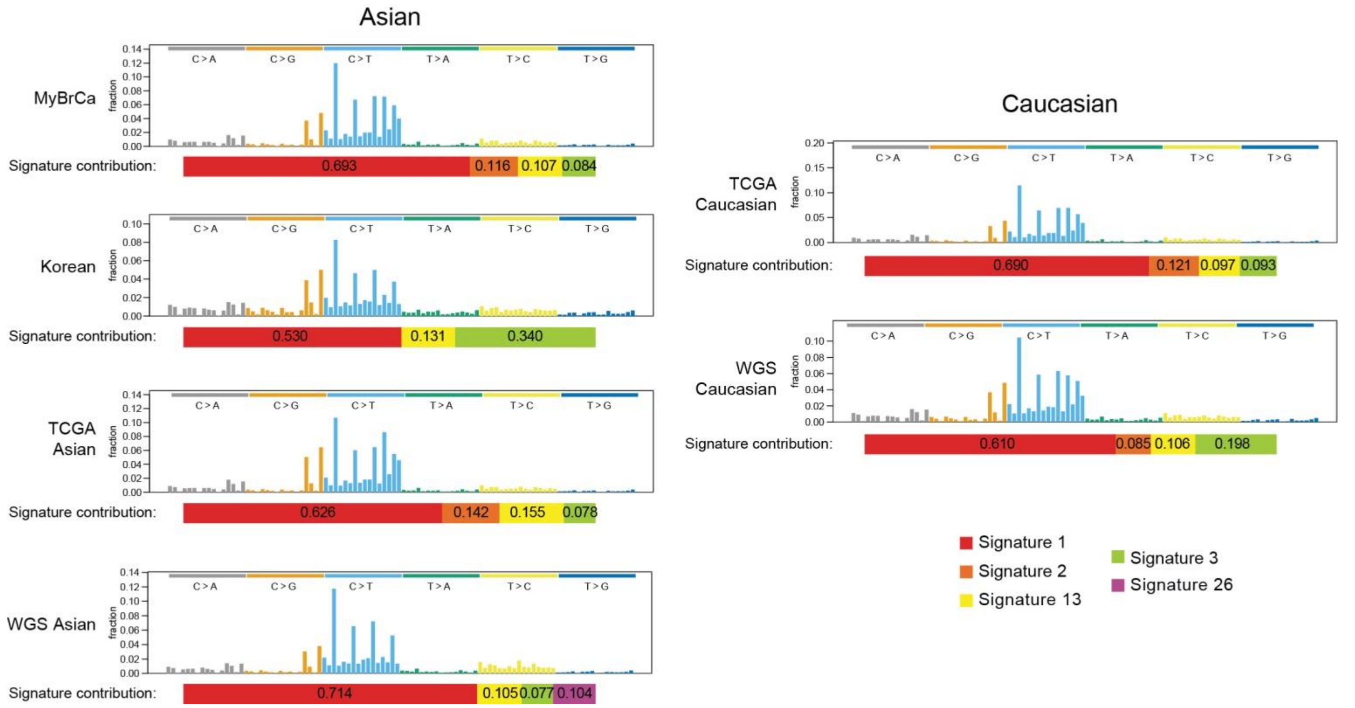

Supplementary Figure 16. Mutational signatures of Asian and Caucasian breast tumours. Mutational spectra of Asian tumours (MyBrCa, Korean, TCGA Asian, WGS Asian) and Caucasian (TCGA Caucasian, WGS Caucasian) generated from all samples from each dataset. Also shown are the relative contributions of the mutational signatures in each data set (bottom bars).

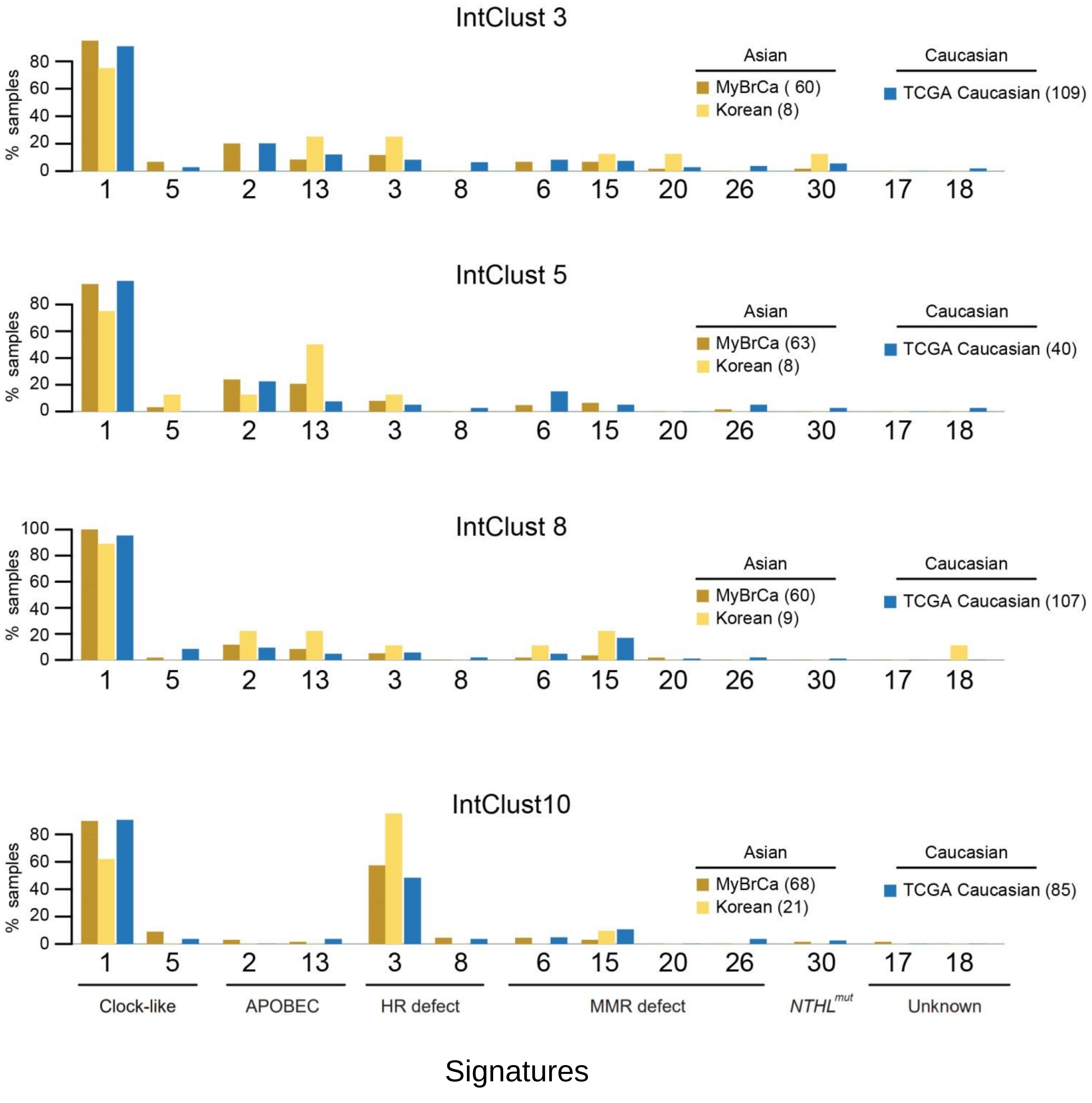

Supplementary Figure 17. Mutational signatures of Asian and Caucasian breast tumours. Prevalence of mutational signatures in Asian (MyBrCa and Korea) and Caucasian (TCGA Caucasian) tumours, limited to IntClusts 3, 5, 8 and 10.

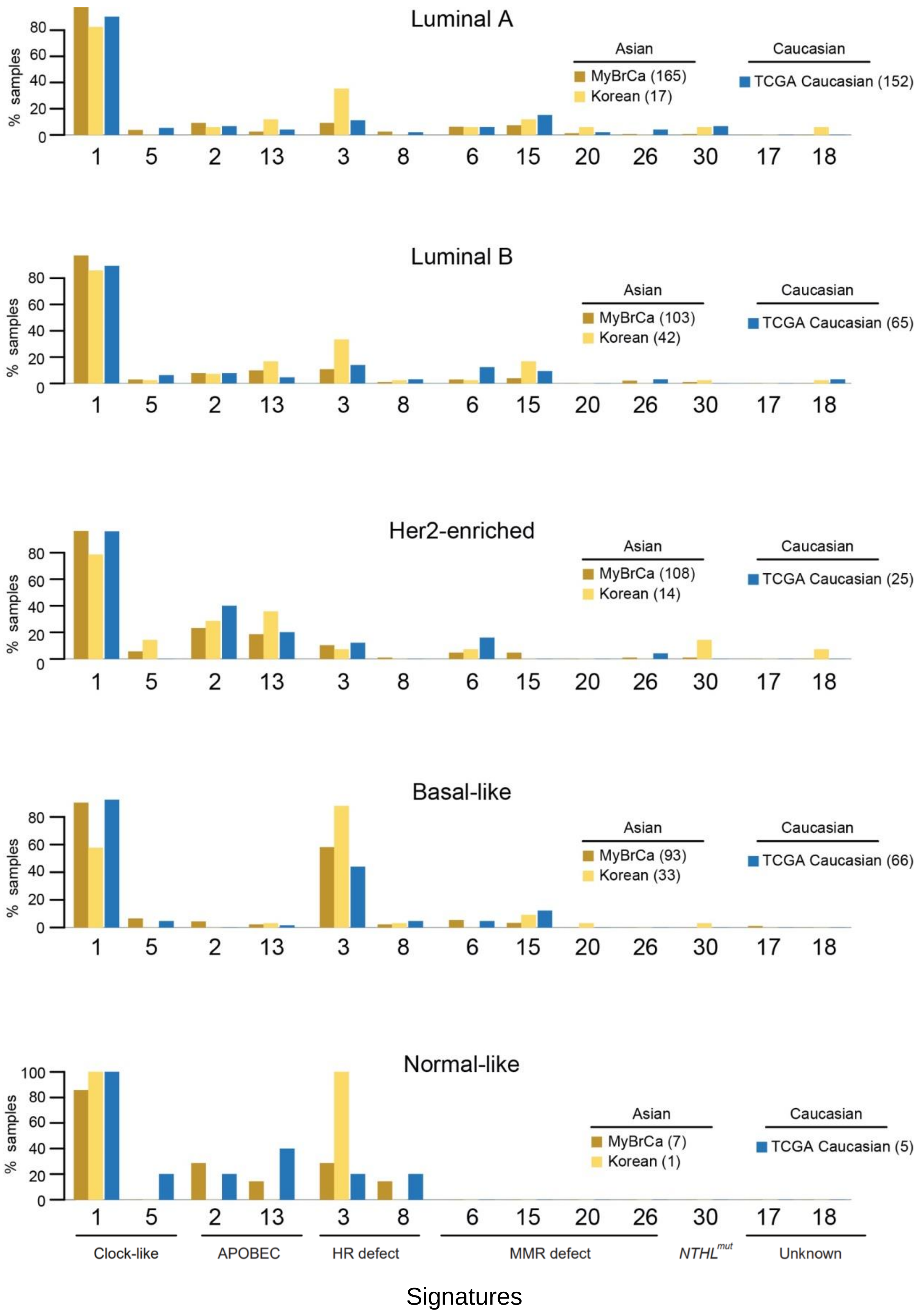

Supplementary Figure 18. Mutational signatures of Asian and Caucasian breast tumours. Prevalence of mutational signatures in Asian (MyBrCa and Korea) and Caucasian (TCGA Caucasian) breast tumours, limited PAM50 subtypes.

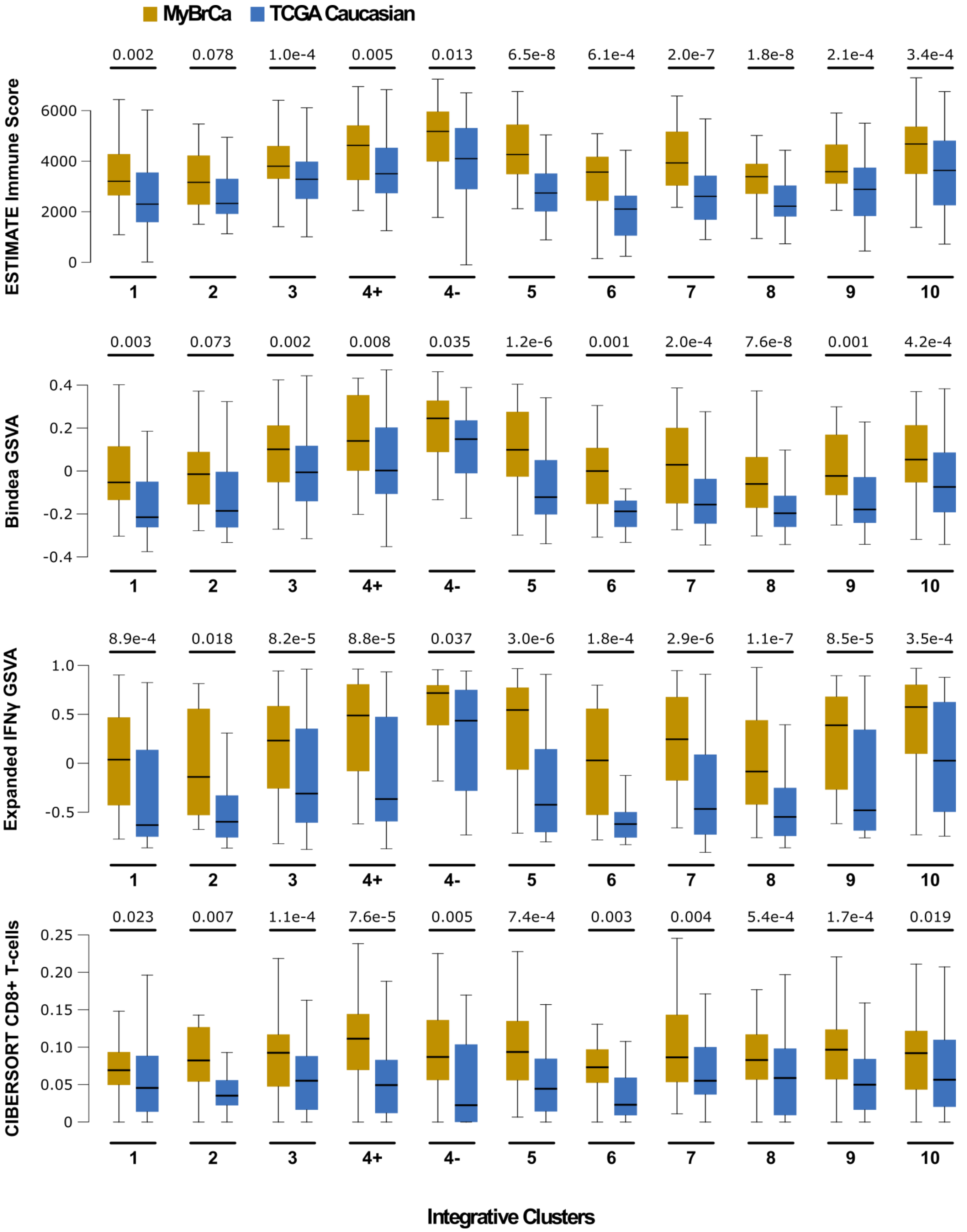

Supplementary Figure 19. Comparison of immune scores across Integrative Cluster subtypes between MyBrCa samples and Caucasian samples from TCGA. P-values are for two-sided *t*-tests between MyBrCa and Caucasian samples for that specific cluster.

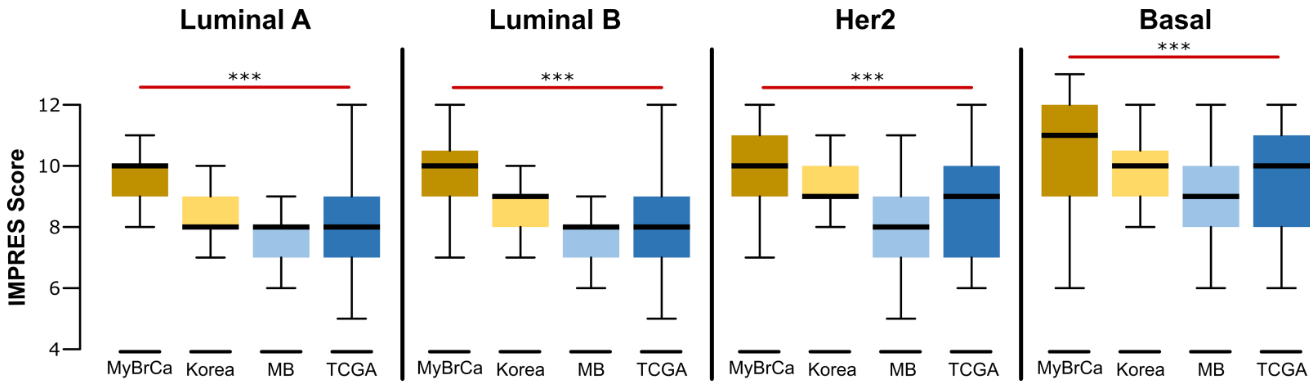

Supplementary Figure 20. Comparison of IMPRES scores across PAM50 subtypes in the MyBrCa, Korean, METABRIC, and TCGA cohorts. Asterisks indicate significant differences between MyBrCa and TCGA samples from a 2-sided t-test ($p<0.001$). Exact p-values, from left to right, are $p<2.2e-16$ (Luminal A), $p<2.2e-16$ (Luminal B), $p=7.6e-11$ (Her2), and $p=9.5e-8$ (Basal). Outliers not shown.

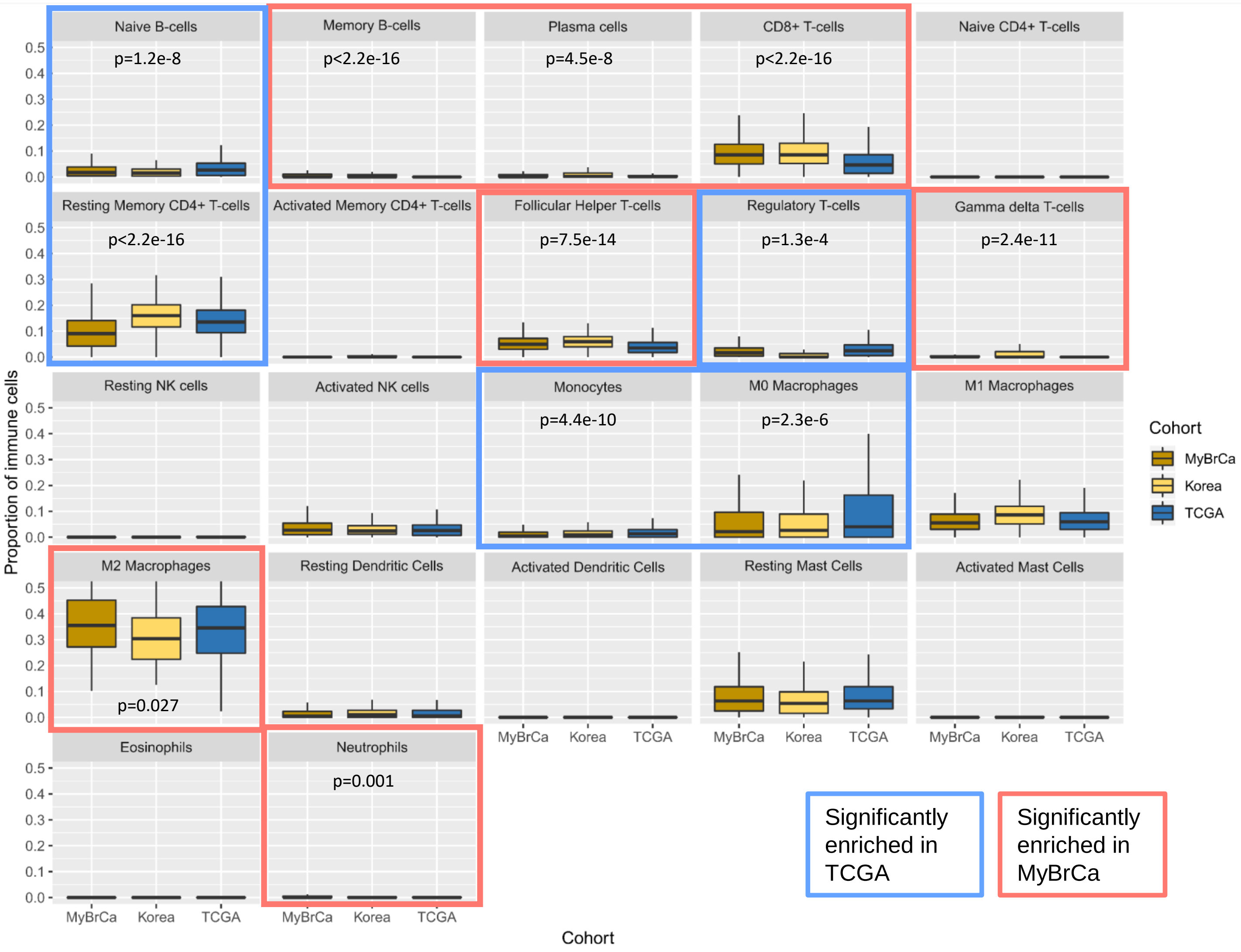

Supplementary Figure 21. CIBERSORT analysis of MyBrCa, Korean, and TCGA BRCA samples. Highlighted boxes indicate immune cell types that were significantly enriched in either TCGA or MyBrCa relative to the other using a 2-sided t-test (p<0.05), with the exact p-values shown. Outliers not shown. The boxes in box plots indicate 25th percentile, median, and 75th percentile, while whiskers show the maximum and minimum values within 1.5 times the inter-quartile range from the edge of the box.

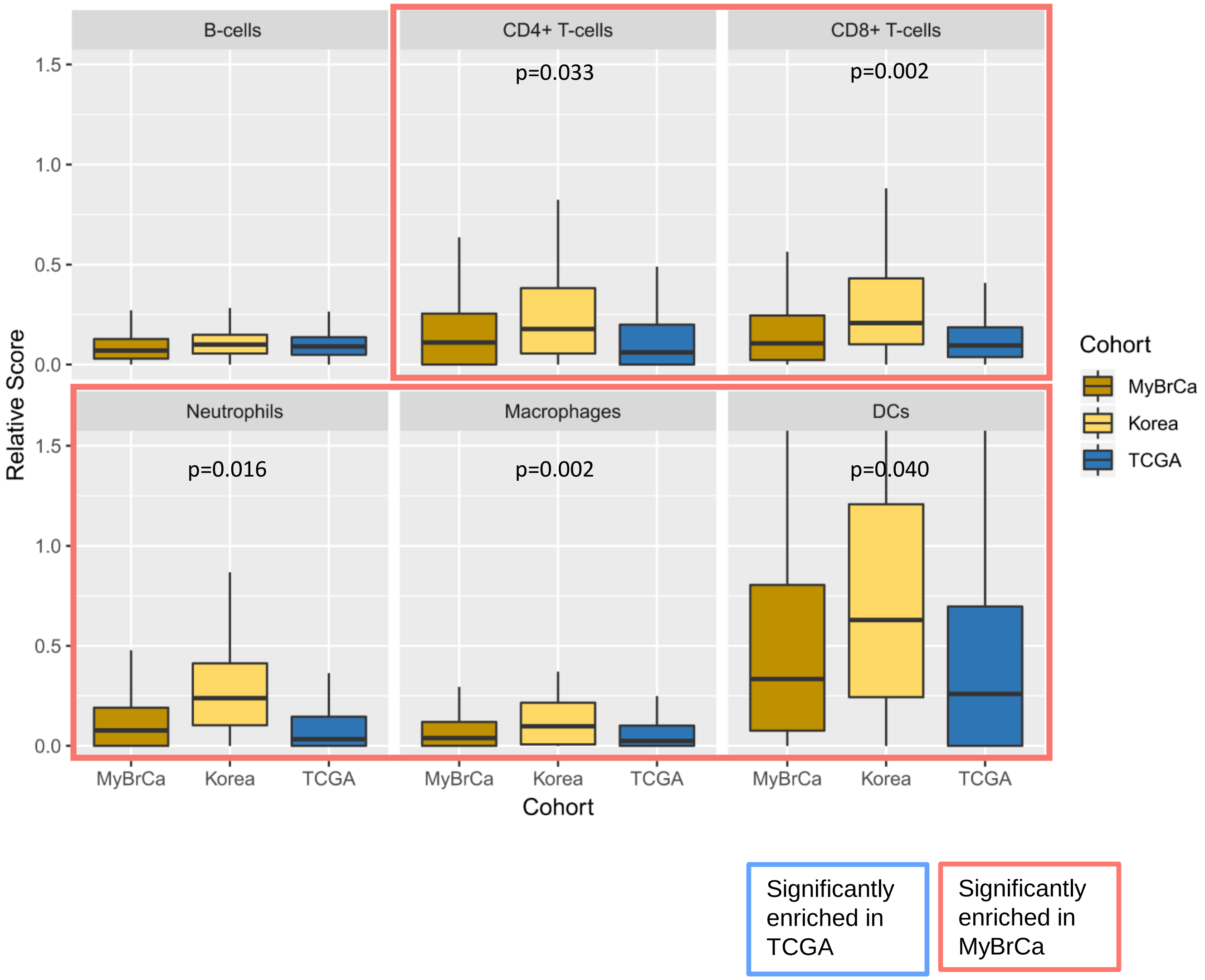

Supplementary Figure 22. TIMER analysis of MyBrCa, Korean, and TCGA BRCA samples. Highlighted boxes indicate immune cell types that were significantly enriched in either TCGA or MyBrCa relative to the other using a 2-sided *t*-test ($p<0.05$), with the exact p-values shown. Outliers not shown.

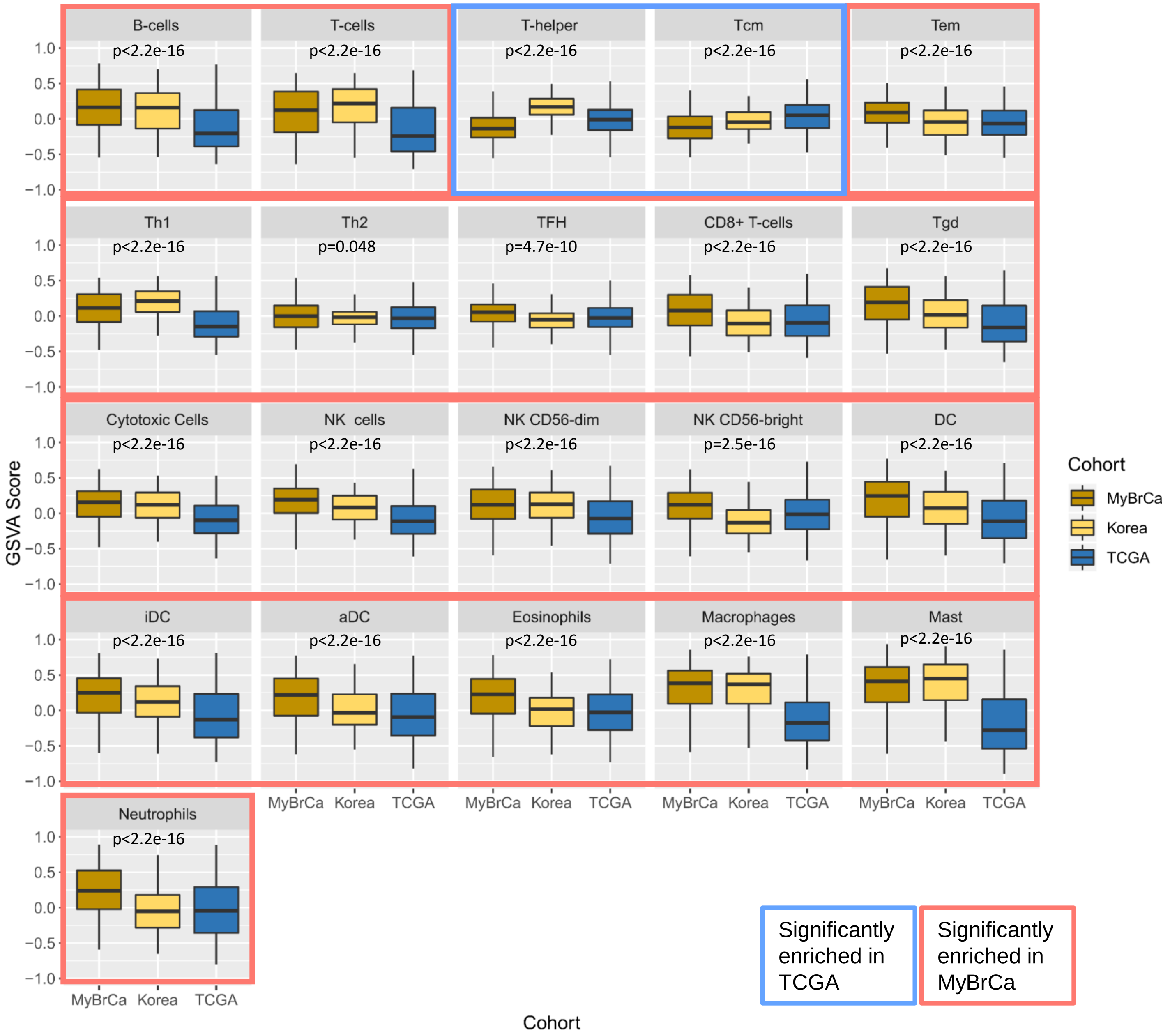

Supplementary Figure 23. GSVA analysis of MyBrCa, and TCGA BRCA samples using gene sets for individual immune cell types from Bindea et al (2013)[6]. Highlighted boxes indicate immune cell types that were significantly enriched in either TCGA or MyBrCa relative to the other using a 2-sided t-test (p<0.05), with the exact p-values shown. Outliers not shown.

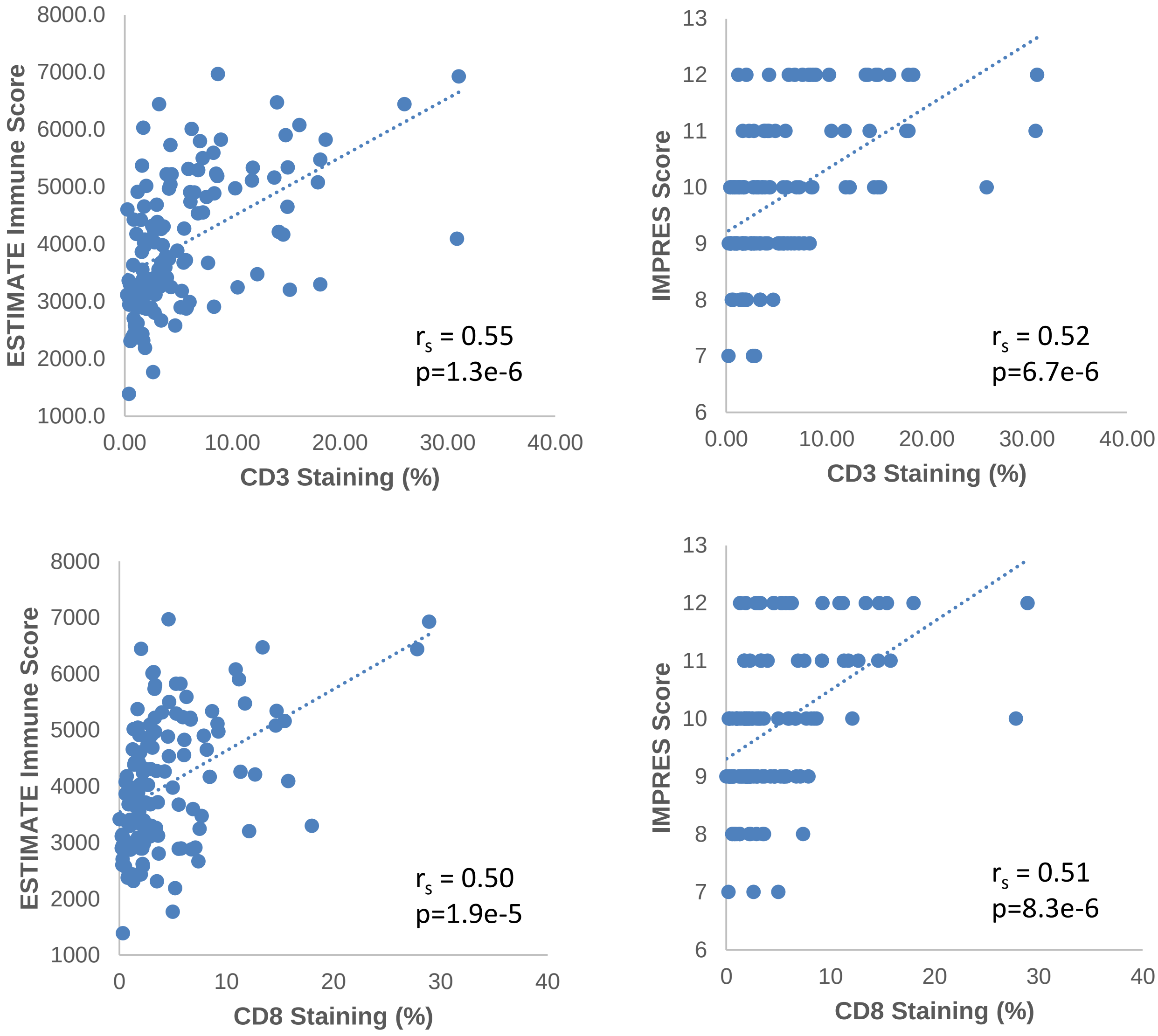

Supplementary Figure 24. Comparison of ESTIMATE (left) and IMPRES (right) immune scores versus CD3 (top) and CD8 (bottom) IHC staining in the MyBrCa cohort (n=124). Trendlines show linear regression. P-values indicated are for Spearman's correlation coefficient (r_s).

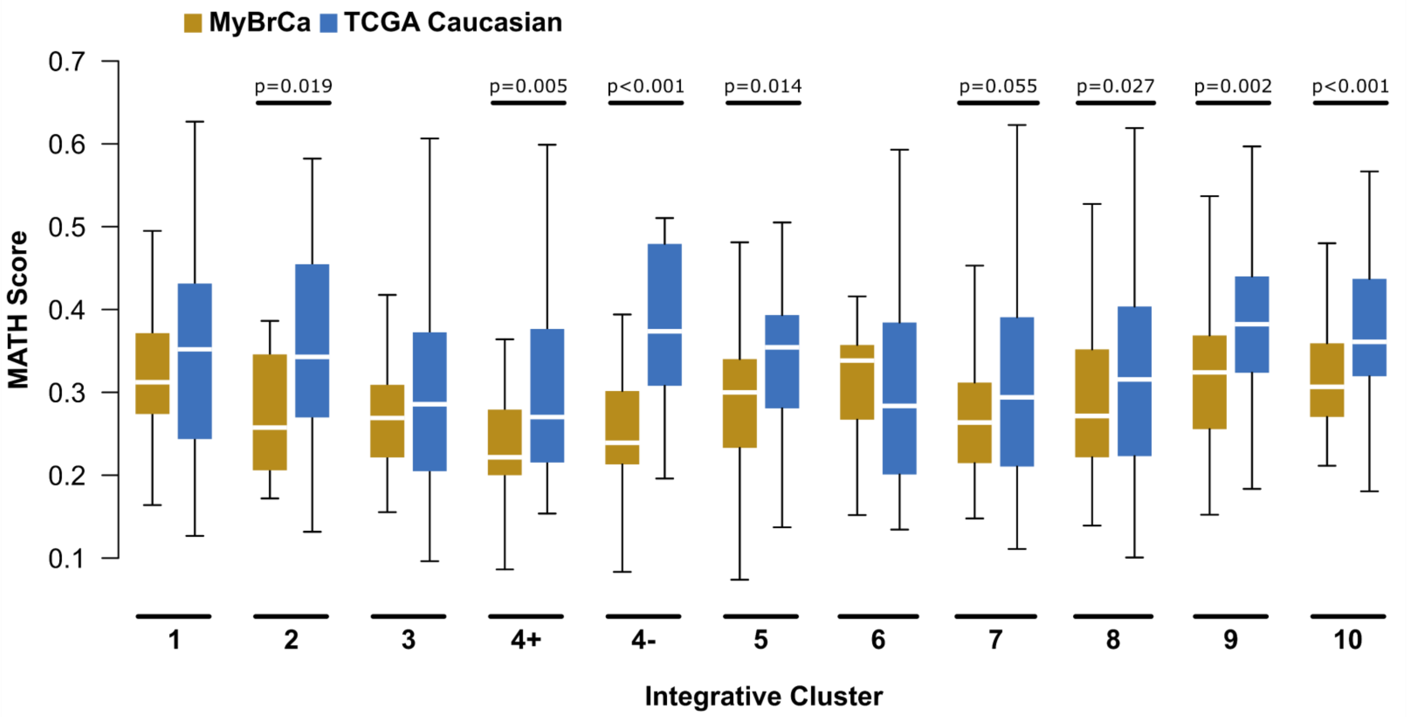

Supplementary Figure 25. Comparison of tumour heterogeneity between the MyBrCa and TCGA cohorts across Integrative Cluster subtypes. Tumour heterogeneity is quantified here using Mutant Allele Tumour Heterogeneity (MATH) scores. P-values shown are for 2-sided student's t-tests. Exact p-values for IC4- and IC10 are p=2.3e-5 and p=5.6e-6, respectively.

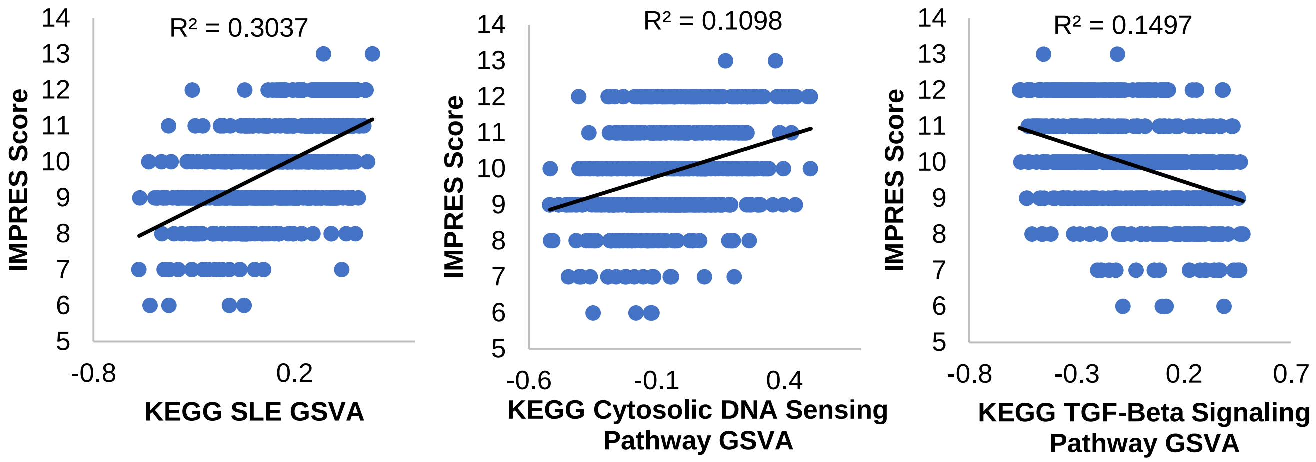

Supplementary Figure 26. Association of IMPRES scores with GSVA Scores for the KEGG Systemic Lupus Erythematosus pathway, KEGG Cytosolic DNA-sensing pathway, and KEGG TGF-Beta Signaling Pathway. Figures shown are for the MyBrCa cohort samples only.

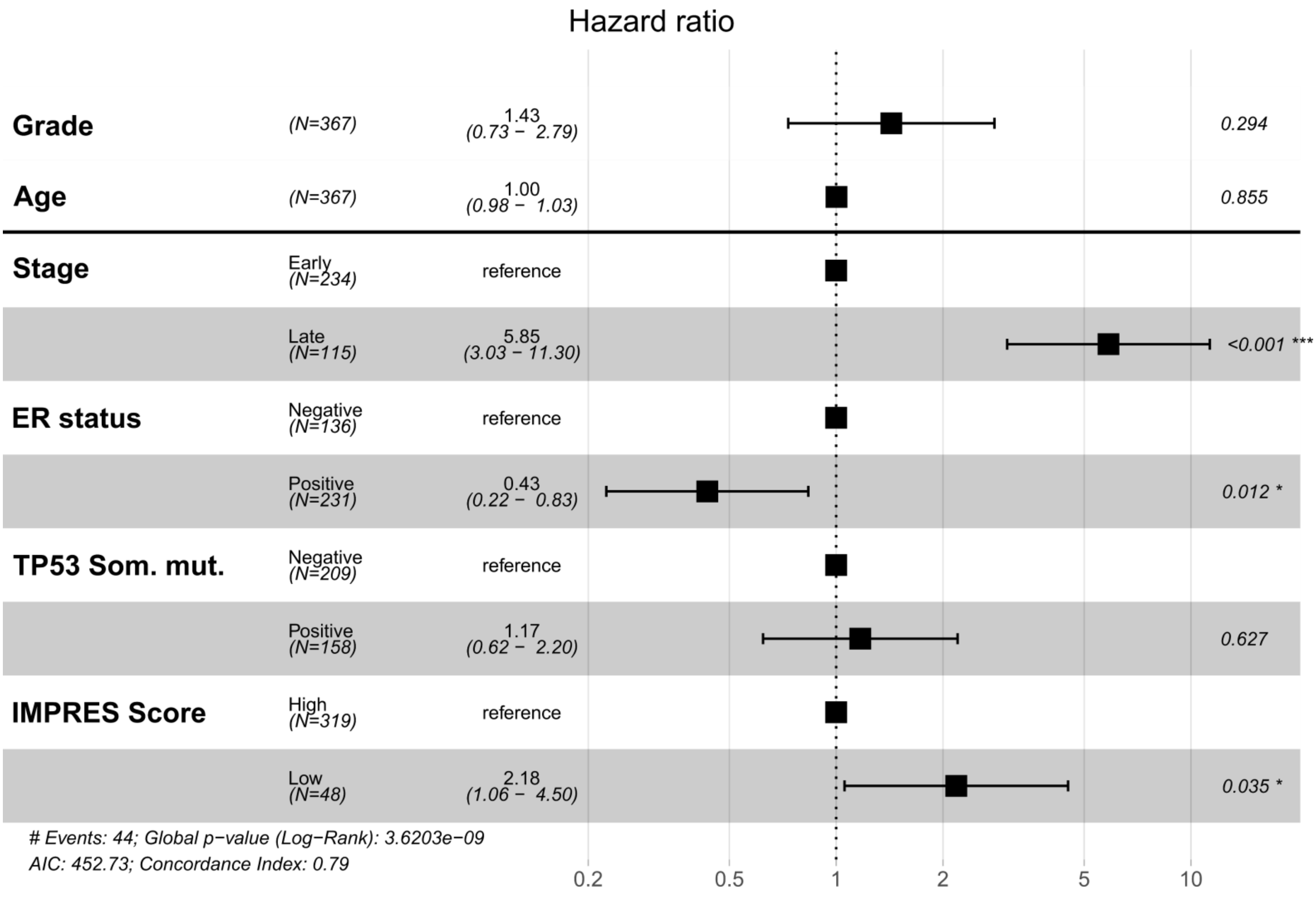

Supplementary Figure 27. Cox Proportional Hazard model of overall survival for MyBrCa patients. Names of the included variables are bolded on the left, p-values of the 2-tailed Z-test for each individual variable are indicated on the right. Only patients with more than 2 years of follow up data were included (n=367). Error bars indicate 95% confidence intervals of the hazard ratio.

Supplementary Table 1. Cohort characteristics. Statistical significance determined with a chi-square test, excluding N/As.

	MyBrCa	TCGA	Statistical significance
Subjects (n)	560	1,097	
Patient age (yr)	53.4 ± 11.7	58.4 ± 13.2	p<1e-4
Parity (n)	3.2 ± 1.6	NA	
**Menopausal status (n(%))**			p<1e-5
Pre-menopausal	173 (30.9)	229 (20.9)	p<1e-5
Post-menopausal	243 (43.4)	705 (64.3)	p<1e-5
Peri-menopausal	-	39 (3.6)	
N/A	144 (25.7)	124 (11.3)	
**Clinical subtype (n(%))**			p<1e-5
ER+/HER2-	274 (48.9)	597 (54.4)	p<1e-5
ER+/HER2+	75 (13.4)	142(12.9)	p=0.51
ER-/HER2+	85 (15.2)	42 (3.8)	p<1e-5
TNBC	116 (20.7)	174 (15.9)	p=0.17
N/A	10 (1.8)	142 (12.9)	
**TNM stage (n(%))**			p=3.3e-4
0	19 (3.4)	-	
I	96 (17.1)	183 (16.7)	p=0.68
II	256 (45.7)	621 (56.6)	p=1.1e-4
III	168 (30.0)	249 (22.7)	p=4.9e-4
IV	17 (3.0)	20 (1.8)	p=0.10
N/A	4 (0.7)	24 (2.2)	
**Histology subtype (n(%))**			p<1e-5
Lobular Carcinoma	18 (3.2)	203 (18.5)	p<1e-5
Ductal Carcinoma	520 (92.9)	784 (71.5)	p<1e-5
Other	19 (3.4)	109 (9.9)	p<1e-5
N/A	3 (0.5)	1 (0.1)	
**Race (n(%))**			
Asian	543 (97)	61 (5.6)	p<1e-5
- Malaysian (Chinese)	498 (89)	-	
- Malaysian (Malay)	26 (4.6)	-	
- Malaysian (Indian)	19 (3.4)	-	
Black	-	183 (16.7)	
White (Hispanic)	-	34 (3.1)	
White (Non-Hispanic)	-	656 (59.8)	
Other	9 (1.6)	72 (6.6)	
N/A	8 (1.4)	91 (8.3)	

Supplementary Table 2. Driver gene analysis on Asian and Caucasian ER+ tumours. Percentage of samples carrying the mutated driver genes, as well as ONC and TSG scores (see Methods) for Asian and Caucasian ER+ tumours. Rows in orange are genes that have been identified as cancer drivers by Integrative Onco Genomics[7] (see Methods).

	ER+					
gene	%Asian	%Caucasian	ONC(Asian)	ONC(Caucasian)	TSG(Asian)	TSG(Caucasian)
AKT1	4.3	1.7	87.0	69.2	0.0	7.7
ANKRD11	0.7	1.7	25.0	6.3	0.0	37.5
ANKRD12	0.9	1.8	20.0	6.7	20.0	33.3
ARHGAP35	0.7	1.5	25.0	8.3	50.0	41.7
ARID1A	4.3	3.5	4.2	3.4	79.2	62.1
ATP2B2	1.3	1.4	14.3	7.7	0.0	7.7
ATR	2.6	2.2	6.7	5.0	6.7	25.0
BRCA1	0.9	1.0	20.0	12.5	20.0	0.0
BRCA2	1.9	2.2	10.0	5.3	40.0	26.3
C1orf173	0.7	1.3	25.0	7.7	0.0	38.5
CASZ1	0.4	2.4	0.0	10.5	50.0	26.3
CBFB	4.8	4.0	3.8	6.5	80.8	74.2
CDH1	5.6	9.2	3.3	2.6	80.0	76.6
CDH24	0.4	1.0	50.0	12.5	0.0	62.5
CDKN1B	0.9	1.0	0.0	9.1	80.0	54.5
CHD4	1.9	2.7	10.0	9.1	10.0	22.7
CHD6	0.9	2.7	20.0	4.5	0.0	27.3
CREBBP	1.9	1.7	10.0	7.1	0.0	14.3
CSMD1	4.1	5.0	4.0	8.7	24.0	4.3
CTCF	2.0	2.8	9.1	18.2	36.4	31.8
DAZAP1	0.6	1.0	0.0	10.0	33.3	50.0
DYSF	2.2	3.5	7.7	2.9	7.7	20.6
ERBB2	1.7	2.6	11.1	30.4	0.0	0.0
FBN3	1.5	2.8	12.5	4.2	0.0	20.8
FER1L6	1.7	1.3	11.1	8.3	0.0	8.3
FLT4	0.6	1.3	33.3	8.3	0.0	8.3
FOXA1	0.9	3.1	20.0	30.0	20.0	10.0
FSIP2	1.7	2.8	11.1	4.3	11.1	21.7
GATA3	15.6	14.2	1.1	3.6	93.3	85.7
GIGYF2	0.9	1.2	20.0	11.1	0.0	55.6
GPR98	3.4	3.9	4.5	2.9	4.5	17.6
GPS2	0.4	1.4	0.0	0.0	50.0	72.7
HECTD4	0.4	2.3	50.0	4.0	0.0	36.0
HELZ	0.7	1.8	20.0	5.3	20.0	31.6
HSPG2	1.7	2.3	11.1	4.3	22.2	26.1
JAG1	1.1	1.9	16.7	6.7	16.7	33.3
JAK1	0.2	1.2	0.0	9.1	0.0	72.7
JMJD1C	1.1	2.2	16.7	5.0	16.7	35.0
KDM6A	0.7	2.3	25.0	5.6	0.0	38.9
KIAA0947	0.6	1.3	33.3	7.1	66.7	35.7
KMT2A	1.5	3.0	11.1	4.0	44.4	32.0
KMT2B	1.3	1.8	14.3	5.6	14.3	38.9
KMT2C	4.7	8.5	3.6	1.3	75.0	60.3
LRP1	1.5	3.3	12.5	3.4	12.5	31.0
MAP2K4	2.4	4.9	15.4	10.5	30.8	52.6
MAP3K1	8.4	9.9	5.6	3.8	53.7	77.4
MAP3K4	1.1	2.2	16.7	5.9	0.0	35.3
MED23	3.4	1.4	5.6	8.3	27.8	58.3
MLLT4	1.7	2.2	11.1	5.3	44.4	36.8
MYB	0.6	1.9	33.3	5.9	66.7	47.1
NACAD	0.4	1.5	50.0	7.1	0.0	35.7
NAV3	0.7	2.1	20.0	5.6	20.0	38.9
NCOR1	2.0	5.5	9.1	4.4	63.6	44.4
NF1	4.1	3.2	4.5	3.8	31.8	30.8
NIPBL	1.9	1.8	10.0	5.6	20.0	22.2
NOTCH2	0.9	3.0	16.7	3.8	0.0	26.9
PIK3CA	32.0	35.4	71.6	66.7	1.1	1.9
PTEN	4.3	4.5	4.0	2.9	68.0	62.9
RB1	1.5	2.2	12.5	5.6	50.0	44.4
RUNX1	2.6	3.2	6.7	4.0	46.7	64.0
SACS	0.7	2.1	25.0	4.5	0.0	27.3
SBF1	0.4	1.5	0.0	6.7	0.0	33.3
SETD2	1.7	1.5	11.1	6.7	22.2	40.0
SF3B1	1.5	3.5	12.5	58.6	0.0	0.0
SMCHD1	0.6	1.8	25.0	6.3	0.0	31.3
SPEN	1.5	4.2	11.1	2.0	55.6	46.9
TAF1B	0.2	0.4	0.0	33.3	100.0	33.3
TBX3	3.0	4.2	11.8	5.6	52.9	55.6
TMEM247	0.2	1.0	0.0	18.2	0.0	45.5
TP53	30.7	17.6	12.0	15.0	38.6	32.1
TRIP12	1.5	1.4	11.1	7.1	33.3	35.7
USP9X	1.5	2.3	12.5	5.3	0.0	26.3
VPS13C	3.4	2.4	5.3	4.5	0.0	22.7
WDR52	0.4	1.0	0.0	11.1	0.0	55.6
ZFHX3	1.7	1.9	10.0	5.9	10.0	41.2
ZFP36L1	1.1	1.0	16.7	0.0	83.3	37.5
ZFPM1	1.3	1.3	14.3	9.1	85.7	81.8
ZMYM3	0.7	1.5	20.0	6.7	20.0	33.3
ZNF142	1.1	1.4	16.7	8.3	0.0	41.7
ZNF292	0.7	1.8	25.0	4.8	25.0	23.8

Supplementary Table 3. Driver gene analysis on Asian and Caucasian ER– tumours. Percentage of samples carrying the mutated driver genes, as well as ONC and TSG scores (see Methods) for Asian and Caucasian ER– tumours. Rows in orange are genes that have been identified as cancer drivers by Integrative Onco Genomics[7] (see Methods).

	ER–					
gene	%Asian	%Caucasian	ONC(Asian)	ONC(Caucasian)	TSG(Asian)	TSG(Caucasian)
AKT1	1.2	1.1	50.0	66.7	0.0	0.0
ANKRD11	2.4	2.7	12.5	11.1	0.0	0.0
ANKRD12	1.2	1.1	25.0	0.0	0.0	33.3
ARHGAP35	1.5	2.7	0.0	12.5	16.7	50.0
ARID1A	1.8	1.5	16.7	25.0	16.7	50.0
ATP2B2	3.0	3.1	9.1	16.7	0.0	41.7
ATR	4.2	5.0	5.6	7.7	5.6	0.0
BRCA1	3.6	3.1	7.1	11.1	42.9	55.6
BRCA2	2.7	2.7	11.1	12.5	22.2	62.5
C1orf173	2.1	2.7	14.3	14.3	14.3	14.3
CASZ1	1.2	1.1	0.0	33.3	50.0	0.0
CBFB	0.3	1.1	0.0	33.3	0.0	33.3
CDH1	0.6	1.9	0.0	20.0	0.0	20.0
CDH24	0.0	0.4	0.0	100.0	0.0	0.0
CDKN1B	0.0	0.8	0.0	33.3	0.0	33.3
CHD4	2.4	2.3	10.0	16.7	0.0	16.7
CHD6	2.1	4.6	14.3	8.3	0.0	16.7
CREBBP	3.6	4.6	7.1	7.7	35.7	23.1
CSMD1	5.6	8.4	3.7	4.3	7.4	13.0
CTCF	1.5	0.8	20.0	0.0	40.0	50.0
DAZAP1	0.3	1.5	0.0	25.0	0.0	25.0
DYSF	2.1	5.4	11.1	6.3	11.1	18.8
ERBB2	3.3	1.9	14.3	20.0	0.0	0.0
FBN3	2.1	4.2	14.3	9.1	0.0	27.3
FER1L6	3.3	4.6	8.3	8.3	8.3	41.7
FLT4	1.5	2.7	20.0	10.0	0.0	50.0
FOXA1	0.3	1.9	0.0	14.3	0.0	14.3
FSIP2	5.6	4.6	5.0	6.7	10.0	6.7
GATA3	1.5	0.4	0.0	0.0	80.0	100.0
GIGYF2	2.1	3.4	11.1	11.1	11.1	11.1
GPR98	6.2	4.6	4.5	7.1	4.5	35.7
GPS2	0.3	0.8	100.0	0.0	0.0	50.0
HECTD4	3.0	3.8	10.0	8.3	0.0	16.7
HELZ	2.4	1.9	10.0	16.7	10.0	0.0
HSPG2	3.6	7.3	7.1	5.3	7.1	5.3
JAG1	2.7	1.5	11.1	25.0	11.1	0.0
JAK1	1.2	0.8	16.7	0.0	33.3	50.0
JMJD1C	2.1	2.3	12.5	14.3	0.0	0.0
KDM6A	0.9	2.7	25.0	12.5	25.0	25.0
KIAA0947	1.5	2.7	16.7	12.5	33.3	12.5
KMT2A	2.7	1.1	11.1	33.3	11.1	0.0
KMT2B	1.2	1.1	25.0	33.3	0.0	33.3
KMT2C	5.9	8.0	7.7	3.8	30.8	38.5
LRP1	3.0	7.3	10.0	4.8	0.0	9.5
MAP2K4	0.3	1.1	0.0	0.0	0.0	66.7
MAP3K1	0.9	2.7	0.0	14.3	0.0	42.9
MAP3K4	2.1	0.4	11.1	0.0	11.1	0.0
MED23	1.2	1.1	25.0	33.3	25.0	33.3
MLLT4	2.1	3.4	14.3	10.0	28.6	20.0
MYB	1.2	0.8	16.7	50.0	0.0	0.0
NACAD	0.6	0.8	50.0	50.0	0.0	0.0
NAV3	2.7	4.6	9.1	8.3	9.1	0.0
NCOR1	3.6	3.8	7.1	10.0	42.9	0.0
NF1	5.3	5.4	4.5	6.7	36.4	53.3
NIPBL	3.9	5.0	5.9	7.1	23.5	35.7
NOTCH2	2.7	4.6	11.1	5.6	33.3	27.8
PIK3CA	21.1	11.9	59.8	47.4	2.4	5.3
PTEN	4.5	4.2	5.9	7.7	76.5	69.2
RB1	4.7	9.6	5.9	3.7	64.7	55.6
RUNX1	2.1	1.1	14.3	0.0	42.9	0.0
SACS	2.7	4.6	10.0	7.1	0.0	7.1
SBF1	0.6	0.8	50.0	50.0	0.0	50.0
SETD2	3.0	1.9	10.0	16.7	20.0	16.7
SF3B1	0.6	0.4	50.0	0.0	0.0	0.0
SMCHD1	0.9	3.1	25.0	11.1	0.0	0.0
SPEN	3.3	3.4	7.1	11.1	21.4	22.2
TAF1B	1.5	2.7	20.0	12.5	60.0	62.5
TBX3	1.5	1.1	20.0	25.0	60.0	0.0
TMEM247	0.0	0.4	0.0	50.0	0.0	0.0
TP53	67.4	71.3	22.2	12.0	34.8	46.4
TRIP12	1.2	3.4	14.3	11.1	0.0	22.2
USP9X	2.7	4.2	9.1	9.1	9.1	36.4
VPS13C	3.0	4.2	6.7	7.7	13.3	0.0
WDR52	2.7	5.0	7.7	7.7	7.7	0.0
ZFHX3	2.4	2.3	10.0	12.5	50.0	25.0
ZFP36L1	0.9	1.1	33.3	0.0	33.3	33.3
ZFPM1	0.3	0.4	0.0	0.0	0.0	0.0
ZMYM3	1.8	1.5	16.7	0.0	16.7	75.0
ZNF142	0.3	1.1	0.0	33.3	100.0	0.0
ZNF292	2.4	1.9	10.0	16.7	20.0	0.0

Supplementary Table 4. Germline mutations in MyBrCa and TCGA breast tumours. Table compares the frequency of germline mutations in highly-penetrant risk genes in the MyBrCa and TCGA cohorts. Significance determined by chi-square tests.

Gene	Total MyBrCa patients	Total MyBrCa carriers	Frequency of carriers in MyBrCa (%)	Total TCGA patients	Total TCGA carriers	Frequency of carriers in TCGA (%)	p-value for MyBrCa vs. TCGA
BRCA1	548	9	1.6	1076	21	2.0	0.66
BRCA2		10	1.8		20	1.9	0.96
PALB2		6	1.1		3	0.3	0.036
ATM		2	0.4		9	0.8	0.27
CHEK2		1	0.2		3	0.3	0.71

Supplementary Table 5. Pathway analysis of MyBrCa versus TCGA tumours. Top 15 most differentially enriched pathways between the MyBrCa and TCGA cohorts as indicated by Gene Set Enrichment Analysis of MSigDB hallmark pathways, ranked by absolute normalized enrichment score (|NES|). Immune system-related gene sets are highlighted in blue.

Rank	Hallmark Pathway	|NES|	p-val	FDR	Higher in
1	Mitotic Spindle	2.061	0	0.012	TCGA
2	Estrogen Response Early	2.016	0	0.009	TCGA
3	UV Response Down	1.953	0	0.018	TCGA
4	Allograft Rejection	1.928	0.004	0.088	MyBrCa
5	TGF Beta Signaling	1.818	0.011	0.041	TCGA
6	Notch Signaling	1.762	0.007	0.051	TCGA
7	Apical Surface	1.753	0.004	0.047	TCGA
8	Wnt Beta Catenin Signaling	1.716	0.012	0.051	TCGA
9	Complement	1.709	0.019	0.289	MyBrCa
10	Oxidative Phosphorylation	1.578	0.092	0.418	MyBrCa
11	Inflammatory Response	1.565	0.100	0.339	MyBrCa
12	Interferon Gamma Response	1.551	0.113	0.292	MyBrCa
13	Interferon Alpha Response	1.542	0.117	0.253	MyBrCa
14	Hedgehog Signaling	1.526	0.047	0.139	TCGA
15	Adipogenesis	1.525	0.061	0.239	MyBrCa

Supplementary Table 6. Regression analysis of IMPRES score in the MyBrCa and TCGA cohorts (n=1657) using a multiple linear model to control for clinical variables that differ between cohorts. Asterisks indicate the level of significance of two-sided t-tests (Pr(>|t|)) for each variable (*< 0.05; **<0.01; ***<0.001).

	Variable	Est. Coefficient	Std. Error	t-value	Pr(>|t|)	Sig.
Age	Age	-0.001	0.004	-0.306	0.760	
Stage (Relative to Stage 1)	Stage 0	-0.964	0.577	-1.671	0.095	.
	Stage 2	-0.006	0.120	-0.048	0.962	
	Stage 3	-0.025	0.138	-0.181	0.856	
	Stage 4	0.093	0.388	0.240	0.810	
Histological subtype (Relative to IDC)	ILC	0.154	0.132	1.162	0.246	
	Other	-0.214	0.170	-1.261	0.208	
	DCIS	1.210	0.860	1.407	0.160	
**Menopausal status** (Relative to Pre-menopausal)	Post-menopausal	-0.070	0.114	-0.611	0.541	
	**Peri-menopausal**	**-0.758**	**0.270**	**-2.811**	**0.005**	**
**Tumour content (relative to <70%)**	**Tumour content 70+%**	**-0.520**	**0.162**	**-3.209**	**0.001**	**
**IHC**	**HR+**	**-1.022**	**0.106**	**-9.624**	**<2e-16**	***
	HER2+	-0.167	0.110	-1.521	0.129	
**Ethnicity** (Relative to Asian)	**Caucasian**	**-1.013**	**0.142**	**-7.143**	**0.000**	***
	**Other**	**-0.742**	**0.172**	**-4.326**	**0.000**	***

Adjusted R-squared: 0.233
Model p-value: <2e-16

Supplementary Table 7. Regression analysis of IMPRES score in the MyBrCa cohort (n=340) using a multiple linear model across available clinical and demographic data. Asterisks indicate the level of significance of two-sided t-tests (Pr(>|t|)) for each variable (*< 0.05; **<0.01; ***<0.001).

	Variable	Est. Coefficient	Std. Error	t-value	Pr(>|t|)	Sig.
**Integrative Cluster Subtype**	IntClust 1	-0.131	0.341	-0.383	0.702	
(Relative to IntClust 3)	IntClust 2	0.281	0.464	0.606	0.545	
	IntClust 4+	0.329	0.319	1.032	0.303	
	**IntClust 4-**	**1.013**	**0.348**	**2.916**	**0.004**	**
	IntClust 5	0.335	0.304	1.101	0.272	
	IntClust 6	0.091	0.421	0.215	0.830	
	**IntClust 7**	**0.658**	**0.310**	**2.126**	**0.034**	*
	IntClust 8	0.012	0.291	0.04	0.968	
	**IntClust 9**	**0.644**	**0.318**	**2.029**	**0.043**	*
	**IntClust 10**	**1.007**	**0.312**	**3.227**	**0.001**	**
**Histological subtype**	Histology - IDC	-0.198	0.587	-0.338	0.735	
(Relative to DCIS)	Histology - ILC	-0.701	0.768	-0.913	0.362	
	Histology - Other	-0.560	0.978	-0.572	0.567	
**Clinical metrics**	Grade	0.185	0.149	1.242	0.215	
	Stage (AJCC6)	0.108	0.105	1.028	0.305	
	Size	-0.019	0.051	-0.383	0.702	
	Tumor Content	0.000	0.004	-0.005	0.996	
	Neoadjuvant treatment	-0.309	0.413	-0.748	0.455	
**Physical metrics**	**Age**	**-0.015**	**0.008**	**-2.012**	**0.045**	*
	Weight	-0.052	0.098	-0.533	0.594	
	Height	3.350	7.421	0.451	0.652	
	BMI	0.118	0.243	0.485	0.628	
**Demographic factors**	Ethnicity – Indian[1]	-0.214	0.441	-0.486	0.628	
[1]Relative to Chinese	Ethnicity - Malay[1]	-0.238	0.382	-0.621	0.535	
[2]Relative to Income <5,000	Ethnicity - Other[1]	-0.250	0.530	-0.472	0.637	
	Age of menarche	0.057	0.055	1.041	0.299	
	Family history of cancer	0.173	0.181	0.953	0.341	
	Income >10,000[2]	0.108	0.230	0.471	0.638	
	Income 5-10,000[2]	0.042	0.180	0.231	0.817	
	Education	-0.167	0.130	-1.289	0.198	
	Regular alcohol drinker	0.064	0.202	0.316	0.752	

Adjusted R-squared: 0.0674
Model p-value: 0.0047

Supplementary Table 8. Linear regression analysis of IMPRES score in the MyBrCa cohort (n=340) using available molecular data, adjusting for age and subtype. Asterisks indicate the level of significance of two-sided t-tests (Pr(>|t|)) for each variable (*< 0.05; **<0.01; ***<0.001).

	Variable	Est. Coefficient	Std. Error	t-value	Pr(>|t|)	Sig.
**Significant variables from clinical-demographic linear model** [a]Relative to IntClust 3	IntClust 1[a]	0.096	0.295	0.324	0.746	
	IntClust 2[a]	0.521	0.379	1.374	0.170	
	IntClust 4+[a]	0.472	0.292	1.617	0.107	
	**IntClust 4-[a]**	**0.826**	**0.289**	**2.859**	**0.004**	**
	**IntClust 5[a]**	**0.622**	**0.266**	**2.341**	**0.020**	*
	IntClust 6[a]	0.258	0.350	0.737	0.462	
	**IntClust 7[a]**	**0.768**	**0.268**	**2.868**	**0.004**	**
	IntClust 8[a]	0.180	0.254	0.710	0.478	
	**IntClust 9[a]**	**0.948**	**0.287**	**3.305**	**0.001**	**
	**IntClust 10[a]**	**1.197**	**0.290**	**4.121**	**0.000**	***
	Age	0.002	0.006	0.363	0.717	
**Germline mutations** [1]Relative to A3B deletion - Homozygous	Germline mutation in HR pathway	-0.055	0.236	-0.232	0.817	
	A3B deletion - Heterozygous[1]	-0.234	0.181	-1.295	0.196	
	A3B deletion - Normal[1]	-0.345	0.189	-1.827	0.068	.
**Somatic mutations**	Tumor Mutation Burden	0.000	0.001	0.347	0.729	
	TP53 somatic mutations	-0.037	0.144	-0.260	0.795	
**Mutational signatures**	Signature 1	0.343	0.351	0.977	0.329	
	Signature 2	0.553	0.636	0.868	0.386	
	**Signature 3**	**1.281**	**0.472**	**2.716**	**0.007**	**
	Signature 13	0.559	0.715	0.782	0.434	
**Tumour heterogeneity**	**Log PyClone Clusters**	**-0.252**	**0.079**	**-3.179**	**0.002**	**
	MATH	-0.795	0.883	-0.901	0.368	
**Neoantigens**	**HLA A maximum binding affinity**	**-0.005**	**0.002**	**-2.898**	**0.004**	**
	**HLA A,B,C maximum binding affinity**	**0.009**	**0.003**	**2.776**	**0.006**	**
	HLA A neoantigen count	0.000	0.008	-0.023	0.981	
	HLA A,B,C neoantigen count	0.004	0.005	0.672	0.502	

Adjusted R-squared: 0.1324
Model p-value: 2.214e-8

Supplementary Table 9. Pathway analysis of IMPRES high- versus low-scoring tumours. Pathway analysis comparing MyBrCa samples in the top quartile of IMPRES scores to samples in the bottom quartile reveals significant differences in the SLE pathway, cytosolic DNA-sensing pathway and TFG-Beta signaling pathway between the two groups.

KEGG Pathway	|NES|	FDR	Enriched in
Systemic Lupus Erythematosus	2.29	0	Top Quartile
Cytosolic DNA-sensing Pathway	2.02	$3X10^{-4}$	Top Quartile
TGF-Beta Signaling Pathway	1.79	0.04	Bottom Quartile

Supplementary Table 10. Comparison of Her2 positivity by different methods. Concordance between Her2 Positivity by IHC with ERBB2 copy number by sWGS, ERBB2 expression by RNASeq (via mixtools k=2 clusters), and PAM50 Her2 subtype classification. Discordant samples are marked in red.

	ERBB2 CNA (sWGS)		ERBB2 Expression (RNASeq)		PAM50 Her2	
	**Del/Loss/Neutral**	**Gain/Amp**	**Low**	**High**	**No**	**Yes**
**HER2- (IHC)**	356	27	376	7	341	25
**HER2+ (IHC)**	20	135	45	112	56	97
**Total**	538		540		519	
**Discordant**	47		52		81	
**Concordance rate (%)**	91.3		90.4		84.4	

Supplementary References

1. Kan, Z. *et al.* Multi-omics profiling of younger Asian breast cancers reveals distinctive molecular signatures. *Nat. Commun.* **9**, 1725 (2018).
2. Cancer Genome Atlas Network. Comprehensive molecular portraits of human breast tumours. *Nature* **490**, 61–70 (2012).
3. Pereira, B. *et al.* The somatic mutation profiles of 2,433 breast cancers refines their genomic and transcriptomic landscapes. *Nat. Commun.* **7**, 11479 (2016).
4. Nik-Zainal, S. *et al.* Landscape of somatic mutations in 560 breast cancer whole-genome sequences. *Nature* **534**, 47–54 (2016).
5. Burstein, M. D. *et al.* Comprehensive genomic analysis identifies novel subtypes and targets of triple-negative breast cancer. *Clin. Cancer Res.* **21**, 1688–1698 (2015).
6. Bindea, G. *et al.* Spatiotemporal dynamics of intratumoral immune cells reveal the immune landscape in human cancer. *Immunity* **39**, 782–795 (2013).
7. Gonzalez-Perez, A. *et al.* IntOGen-mutations identifies cancer drivers across tumor types. *Nat. Methods* **10**, 1081–1084 (2013).
