# Supplementary material for: The molecular landscape of Asian breast cancers reveals clinically relevant population-specific differences
Source: Nat Commun. 2020 Dec 22;11:6433. doi: 10.1038/s41467-020-20173-5 (PMC7755902; doi:10.1038/s41467-020-20173-5)
Supplement: Supplementary file 2 — Reporting Summary [file 41467_2020_20173_MOESM2_ESM.pdf]

nature research

Corresponding author(s): Suet-Feung Chin, Soo-Hwang Teo

Last updated by author(s): Oct 20, 2020

Reporting Summary

Nature Research wishes to improve the reproducibility of the work that we publish. This form provides structure for consistency and transparency in reporting. For further information on Nature Research policies, see Authors & Referees and the Editorial Policy Checklist .

Statistics

For all statistical analyses, confirm that the following items are present in the figure legend, table legend, main text, or Methods section.

n/a	Confirmed	
☐	☒	The exact sample size (n) for each experimental group/condition, given as a discrete number and unit of measurement
☐	☒	A statement on whether measurements were taken from distinct samples or whether the same sample was measured repeatedly
☐	☒	The statistical test(s) used AND whether they are one- or two-sided<br>*Only common tests should be described solely by name; describe more complex techniques in the Methods section.*
☐	☒	A description of all covariates tested
☐	☒	A description of any assumptions or corrections, such as tests of normality and adjustment for multiple comparisons
☐	☒	A full description of the statistical parameters including central tendency (e.g. means) or other basic estimates (e.g. regression coefficient) AND variation (e.g. standard deviation) or associated estimates of uncertainty (e.g. confidence intervals)
☐	☒	For null hypothesis testing, the test statistic (e.g. F, t, r) with confidence intervals, effect sizes, degrees of freedom and P value noted<br>*Give P values as exact values whenever suitable.*
☒	☐	For Bayesian analysis, information on the choice of priors and Markov chain Monte Carlo settings
☒	☐	For hierarchical and complex designs, identification of the appropriate level for tests and full reporting of outcomes
☐	☒	Estimates of effect sizes (e.g. Cohen's d, Pearson's r), indicating how they were calculated

Our web collection on statistics for biologists contains articles on many of the points above.

Software and code

Policy information about availability of computer code

Data collection	Standard, open-source tools used in data collection are described in the Methods section. These include the STAR aligner (v. 2.5.3a), featureCounts (v. 1.5.3), RSEM (v1.2.31), bwa-mem (v. 0.7.12), Strelka2, and GATK3 and picard from the Broad Institute.
Data analysis	All computational tools used in data analysis are open-source tools that have been previously published, and are described in the Methods section. These include the limma (v. 3.34.9), genefu (v. 2.14.0), iC10 (v. 1.5), mixtools (v. 1.1), ConsensusClusterPlus (v.1.46), ESTIMATE (v. 1.0.13), GSVA (v. 1.26), QDNASeq (v. 1.24.0), ASCAT (v. 2.5.2), alleleCounter (v. 4.0.1), survival (v. 2.44), and edgeR (v. 3.20.9) packages in R, as well as deConstructSigs, PyClone (v 0.13.1), GSEA (v. 3.0), PolySolver, netMHCpan, netMHC, pVAC-seq, and Oncotator (v. 1.9.9.0). Custom code used to generate the figures in the manuscript have been made available on GitHub at https://github.com/panjw0/MyBrCa_Genomics.

For manuscripts utilizing custom algorithms or software that are central to the research but not yet described in published literature, software must be made available to editors/reviewers. We strongly encourage code deposition in a community repository (e.g. GitHub). See the Nature Research guidelines for submitting code & software for further information.

Data

Policy information about availability of data

All manuscripts must include a data availability statement. This statement should provide the following information, where applicable:

- Accession codes, unique identifiers, or web links for publicly available datasets
- A list of figures that have associated raw data
- A description of any restrictions on data availability

Sequencing data for this study (WES, RNA-seq, and sWGS bam files) are available on the European Genome-phenome Archive under the study accession number EGAS00001004518 (https://ega-archive.org/studies/EGAS00001004518). Access to controlled patient data will require the approval of the Data Access Committee. Publicly available datasets that were included in this study are accessible as follows: TCGA10 and METABRIC5 data are available via the Genomic Data Commons Data Portal and cBioportal, while data from Nik-Zainal et al. (2016) are available from ftp://ftp.sanger.ac.uk/pub/cancer/Nik-ZainalEtAl-560BreastGenomes and data from Zhengyan Kan et al. (2018) are available from https://www.nature.com/articles/s41467-018-04129-4. Other public databases that were accessed include

Reactome (www.reactome.org), the Integrative Onco Genomics database (www.intogen.org), and gnomAD (gnomad.broadinstitute.org). The remaining data are available within the Article, Supplementary Information or available from the authors upon request.

Field-specific reporting

Please select the one below that is the best fit for your research. If you are not sure, read the appropriate sections before making your selection.

- [x] Life sciences
- [ ] Behavioural & social sciences
- [ ] Ecological, evolutionary & environmental sciences

For a reference copy of the document with all sections, see nature.com/documents/nr-reporting-summary-flat.pdf

Life sciences study design

All studies must disclose on these points even when the disclosure is negative.

Sample size

The sample size was set in order to detect somatic mutation frequencies of 3% or above (Lawrence et al. 2015 Nature). With 560 samples, the sample size also enables general and subtype-specific molecular comparisons of breast cancer to similar genomic studies of breast cancer in Western populations such as TCGA and METABRIC.

Data exclusions

Patients were excluded from this study for all genomic and transcriptomic analyses for the following criteria: No corresponding tumour samples (n=5), no corresponding germline samples (n=5) and those who withdrew consent (n=12). Tumour samples were further excluded after clinicopathological review if they were found to be from rare histological subtypes and other breast diseases (n=5). Tumour samples with an average tumour content of <30% (n=50) and those with insufficient DNA (n=8) were excluded from the study. After sequencing, samples that did not reach standard sequencing quality metrics were also excluded. These exclusions were determined before analyses were conducted.

Replication

As this was a large genomics study, each genomic and transcriptomic analyses had more than 500 biological replicates overall, and subgroup analyses such as subtype-specific analyses used subsets of this dataset. Additionally, major findings on the increased prevalence of Her2 subtype and TP53 mutations, as well as the enriched immune scores, were replicated in other Asian genomic datasets from Korea (Kan et al. 2018, Nik-Zainal et al. 2016) and TCGA (2012).

Randomization

Given the nature of the study as a comparison of multiple cohorts, randomization was not possible. Sample selection followed the patient recruitment criteria of each cohort; in our case, our patients were recruited sequentially as seen in the clinic of a private hospital over several years. Where appropriate, comparisons were adjusted for covariates that are known to be different between the different comparator groups by including the covariates in a linear model (for determining associations between variables) or in a Cox proportional hazard model (for survival analyses).

Blinding

Investigators were blinded to specific patient identities during data analysis, as data analysis used de-identified patient data. Additionally, the data analysis team were not involved in patient recruitment or data collection.

Reporting for specific materials, systems and methods

We require information from authors about some types of materials, experimental systems and methods used in many studies. Here, indicate whether each material, system or method listed is relevant to your study. If you are not sure if a list item applies to your research, read the appropriate section before selecting a response.

Materials & experimental systems

n/a	Involved in the study	
[ ]	[x]	Antibodies
[x]	[ ]	Eukaryotic cell lines
[x]	[ ]	Palaeontology
[x]	[ ]	Animals and other organisms
[ ]	[x]	Human research participants
[x]	[ ]	Clinical data

Methods

n/a	Involved in the study	
[x]	[ ]	ChIP-seq
[x]	[ ]	Flow cytometry
[x]	[ ]	MRI-based neuroimaging

Antibodies

Antibodies used

Antibodies used were anti-CD3 (clone 2GV6, prediluted; Ventana Medical Systems, catalog # 790-4341, lot # E05761), anti-CD4 (clone SP35, prediluted; Ventana Medical Systems, catalog # 790-4423, lot # E05570), anti-CD8 (clone SP57, prediluted; Ventana Medical Systems, catalog # 790-4460, lot # E05777) and anti-PD-1 (clone SP263, prediluted; Ventana Medical Systems, catalog # 790-4905, lot # Y29020) rabbit monoclonal primary antibodies. Antibodies were used as is without further dilution.

Validation

All antibodies used are commercially available and validated by Roche Diagnostics for in vitro diagnostic (IVD) use in sections of normal and neoplastic human tissues, as listed on the manufacturer's website. Specifically, each lot was validated through testing of tonsil, spleen, lymph node, and T-cell lymphoma tissues (anti-CD3), liver and tonsil tissue (anti-CD4), lymphoma tissue (anti-CD8), or NSCLC and SCCHN tumour cells (anti-PD-L1) with the test batch having intensity scores above specific thresholds

(generally 3.5 but depending on the antibody and test tissues) and within 0.5 points of the reference batch. The background intensity was 0.5 or less with no observable background staining on the glass, and the intensity of the negative reagent control slide was 0.5 or less. Reagents also passed visual inspection as being clear and free of turbidity.

Human research participants

Policy information about studies involving human research participants

Population characteristics	The study population was female, Malaysian breast cancer patients aged 23 to 86 seeking treatment at the Subang Jaya Medical Centre, a private hospital within the Kuala Lumpur metropolitan area. Relative to other similar cohorts such as TCGA and METABRIC, our cohort was younger and predominantly Asian. A comparison of cohort characteristics with the TCGA cohort is provided in the paper.
Recruitment	Patients were recruited sequentially as seem in the clinic from a hospital-based cohort over several years by the surgeon treating the patients (CHY). The hospital in point, Subang Jaya Medical Centre, is a private Malaysian hospital within the Kuala Lumpur metropolitan area, where patients tend to be of higher socio-economic status relative to the general population. However, as income and education were not significant variables in any of our analyses, this is unlikely to affect the major findings of our study.
Ethics oversight	The project was reviewed and approved by the Independent Ethics Committee, Ramsay Sime Darby Health Care (reference no: 201109.4 and 201208.1), and written informed consent was given by each individual patient.

Note that full information on the approval of the study protocol must also be provided in the manuscript.
